# Comprehensive characterization of viticultural biomass and its derived biochars: insights into copper sorption potential

**DOI:** 10.1007/s11356-026-37550-0

**Published:** 2026-03-06

**Authors:** Hugo Henaut, Yassine Chafik, Marta Sena-Velez, Benoît Cagnon, Sylvain Bourgerie, Domenico Morabito

**Affiliations:** 1 P2E, UR 1207 - USC INRAé 1328, Université d’Orléans, Orléans, France; 2 CNRS, ICMN, UMR 7374, Université d’Orléans, Orléans, France

**Keywords:** Biochar, Copper remediation, Viticulture, Sorption, Trace elements

## Abstract

The extensive use of copper (Cu) in viticulture has resulted in its high accumulation in soils, threatening ecosystems and soil health. Biochar, widely recognized for its potential in remediating metal-contaminated environments, offers promising sorption properties that strongly rely on both feedstock composition and the final biochar’s characteristics. This study investigates five biochars produced from different vineyard by-products; grape marc (BM), grape seed (BS), exhausted grape seed (BES), pruning (BP1), and pruning subjected to double pyrolysis (BP2). These biochars were comprehensively characterized in terms of physicochemical and structural properties, elemental composition, and potential toxicity. The maximum copper sorption capacities of the biochars ranged from Q_max_ of 39.35 to 134.36 mg Cu^2^⁺g^−1^, depending on their properties, and followed the Langmuir isotherm and pseudo-second-order (PSO) kinetic models (for BM). Fourier-transform infrared spectroscopy (FT-IR) confirmed the presence of surface functional groups such as –OH, –COOH, and aromatic structures. Zeta potential (ZP) analysis indicated a predominantly negative surface charge across a wide pH range. Copper sorption was primarily attributed to π-cation interaction and precipitation mechanisms, while copper desorption assays revealed limited copper release, suggesting strong retention. Scanning electron microscopy (SEM), coupled with energy-dispersive X-ray spectroscopy (EDS), was used to analyze the morphology of the biochar and to verify copper incorporation in the Cu-loaded samples. Given the strong influence of feedstock on metal sorption efficiency, vineyard by-products were characterized by thermogravimetric analysis (TGA), FT-IR, and determination of lignocellulosic composition and physicochemical properties. Biochar from vineyard by-products, particularly grape marc (BM), demonstrated the highest potential, highlighting the valorization of vineyard by-products as a sustainable in situ remediation strategy in vineyard soils, in line with circular economy principles.

**Supplementary Information:**

The online version contains supplementary material available at 10.1007/s11356-026-37550-0.

## Introduction

In viticulture, copper-based compounds have been widely employed as fungicides to control diseases, with a notable focus in fighting *Plasmopara viticola*, the causal agent of downy mildew (Pump et al. 2019). Despite the multiple advancements in disease management, copper-containing fungicides remain widely used for disease control in vineyards. Their extensive reliance has made such compounds the first cause of copper accumulation in agricultural soils (Gonzaga et al. 2020). Copper, a persistent trace element, tends to accumulate predominantly in the topsoil layer (Brunetto et al. 2014), particularly in vineyard ecosystems (Pietrzak and McPhail 2004; Ruyters et al. 2013), due to the high affinity of copper for organic matter, metal oxides, and clay minerals (Cesco et al. 2021). In many vineyards, copper concentration in the soil frequently exceeds the thresholds established by the European Union for safe agricultural practices (Komárek et al. 2010). Elevated copper concentrations pose significant ecological risks, including detrimental effects on soil microflora and microfauna (Mackie et al. 2015). Sensitive organisms such as earthworms (Eijsackers et al. 2005), snails (Snyman et al. 2009), and microbial communities (Zhang et al. 2022) experience profound impacts, leading to disruption in soil health and ecosystem functionality. Moreover, copper contamination poses a risk to groundwater pollution (Komárek et al. 2010). Through runoff processes, copper may also reach aquatic ecosystems (Banas et al. 2010; Babcsányi et al. 2016).

Although copper is an essential micronutrient for plant growth (Rehman et al. 2019), the high copper concentrations found in soils require remediation programs to mitigate further environmental and ecological risks. In situ remediation strategies are crucial to reduce the exorbitant depollution costs associated with soil excavation and external treatment (Liu et al. 2018). Various remediation techniques have been explored, including phytoaccumulation (Melo et al. 2022), microbial remediation based on fungi and/or bacterial application into the soil (Cornu et al. 2017; Palanivel et al. 2023), and the application of biochar. Biochar, a low-cost, carbon-rich, alkaline material, is produced through high-temperature pyrolysis of diverse feedstocks, most commonly plant biomass, under low or no oxygen conditions (Hui 2021). It has been demonstrated that biochar can be used as soil amendment, pollutant remediation agent, and greenhouse gases reduction material (Abhishek et al. 2022). Indeed, biochar plays a significant role in mitigating climate change by sequestering carbon (Xia et al. 2023). For example, the application of biochar derived from grapevine waste in vineyards has been shown to reduce the carbon footprint of wine production by 18 g of CO_2_ equivalent per bottle produced (Rosas et al. 2015).

Due to its unique physicochemical properties, biochar has demonstrated strong capacities for immobilizing trace elements (Abhishek et al. 2022), thereby reducing their bioavailability in soil and plant uptake. Regarding copper remediation, biochar has shown efficacy when used in aqueous solutions (Lee et al. 2017; Katiyar et al. 2021; Zhang et al. 2021) as well as in contaminated soils (Meier et al. 2017; Cao et al. 2019; Tomczyk et al. 2019). Biochar offers multiple mechanisms of trace elements chemisorption: ion exchange, where metal cations in solution (e.g., Cu^2^⁺, Pb^2+^) replace the biochar’s exchangeable cations (e.g., Ca^2^⁺, K⁺, Mg^2^⁺, originally bound to negatively charged sites on the biochar surface); ion complexation, which occurs when metal cations form chemical bonds with the oxygen-containing functional groups present on the biochar surface (e.g., –COOH, –OH), resulting in the formation of inner-sphere surface complexes; metal precipitation, favored by the presence of mineral phases (e.g., carbonates, phosphates) which leads to the formation of insoluble metal precipitates on biochar’s surface (e.g., Cu(OH)₂, CuCO₃); π–cation interaction between positively charged metal cations and the electron-rich π-systems of aromatic carbon structures commonly found in biochar; and finally, electrostatic attraction, based on the non-specific interaction between negatively charged sites on the biochar surface and positively charged metal cations, leading to outer-sphere sorption that is pH-dependent and reversible (Nguyen et al. 2023; Mei et al. 2025; Lin et al. 2025). The limited desorption of trace elements associated with precipitation, π–cation interactions, and complexation (Bandara et al. 2020) highlights the need to quantify the specific sorption mechanisms involved in each biochar in order to optimize their application. Additionally, physisorption, through pore filling mechanism, refers to the physical entrapment of metal ions within the porous structure of the biochar, especially in micropores and mesopores, without the need for specific chemical interactions (Mei et al. 2025).

The sorption capacities of trace elements are influenced by biochar physicochemical properties such as surface functional groups, surface area and porosity, negative charge, mineral content, and cation exchange capacity (Tan et al. 2015; Cao et al. 2019). Consequently, biochar produced from different feedstocks and pyrolysis conditions may exhibit multiple efficiencies and mechanisms for trace elements removal (Bandara et al. 2020). Besides, viticulture generates significant biomass coproducts potentially valuable, such as grape seeds or grape marc, which are often valorized through distillation and the extraction of valuable compounds (Muhlack et al. 2018). However, vine prunings are frequently discarded or burned, contributing to waste and environmental concerns (Sun et al. 2020; Wei et al. 2022). The use of grapevine biomass as feedstocks for biochar production and its use for trace elements removal is an underexplored area of research. Da Silva et al. (2024) demonstrated that grape marc biochar effectively removes copper from aqueous solutions. Similar material was used by Carvalho et al. (2022) for removal of Ni and Zn from industrial effluent and by Jin et al. (2020) for Pb sorption. Similarly, Trakal et al. (2014) reported that biochar made from grape stalks and husks removes Cd and Pb from aqueous solutions, while Li et al. (2018) showed that biochar derived from vine prunings effectively removes trace elements. Finally, Noli et al. (2022) demonstrated that grape marc biochar successfully removes U(IV) in aqueous solution.

This study was motivated by the need to valorize grapevine by-products from the wine industry (such as grape marc, seeds, and vine pruning) and to evaluate their potential as sustainable feedstock for biochar production. The various lignocellulosic compositions of these by-products are expected to result in diverse structural and surface properties, influencing copper sorption efficiency (Meng et al. 2025); our aim is to identify the most suitable feedstock for copper remediation.Ultimately, by leveraging agricultural waste from the wine industry, this research aims to provide a sustainable solution for mitigating copper contamination in vineyard soils while contributing to circular economy practices in viticulture.The specific objectives were as follows: (1) to assess the influence of feedstock origin on the physicochemical properties of the final materials, including surface characteristics, SEM–EDS, FT-IR, and elemental composition of biochar; (2) to investigate the capacity of these biochars to adsorb copper; and (3) to elucidate the mechanisms underlying copper sorption by comparing the behavior of pristine and Cu-loaded biochar derived from grape biomass.

## Material and methods

### Feedstock recovery and biochar production

Four vineyard by-products were recovered and labelled as follows: grape marc (GM), vine pruning (P), grape seed (S), and exhausted grape seeds (EGS) coming from vineyards in the Centre-Val-De-Loire region, France. Biochars were produced by the slow pyrolysis (6 h) of biomass at 700 °C with between 5 and 10°C/min heating rate and labelled as follow: marc’s biochar (BM); seed’s biochar (BS); biochar from exhausted seed (BES); biochar from pruning (BP1); and pruning’s biochar with double pyrolysis (BP2). All the samples were crushed and sieved to 0.5–2-mm prior analysis.

### Feedstock and biochar characterization

#### Feedstock composition and thermogravimetric analysis

The extractives, hemicellulose, cellulose, and lignin content of feedstock were determined following protocol described by Samba et al. (2025). Thermogravimetric analysis (TGA) spectra were obtained by heating feedstock samples using a *NETZSCH STA 449 G5 Jupiter device* from 30 to 750 °C at 20 °C.min^−1^ under argon. The oil content of feedstocks was determined by hexane extraction using the Soxhlet method, following *ISO 734:2023.*

#### Proximate analysis

The pH, electrical conductivity (EC), bulk density, water holding capacity (WHC), and ash content of both feedstock and derived biochar were measured according to Chafik et al. (2024). Moisture content was determined by heating samples at 100 °C overnight and measuring mass loss. Volatile matter was measured by heating samples for 7 min at 950 °C with low oxygen environment. Fixed carbon was calculated as 100 − (Volatile matter + Ash + Moisture). Water retention curves of biochar in a sandy soil (2% w/w) was assessed following *NF EN 13041.* Cation exchange capacity (CEC) was assessed following Munera-Echeverri et al. (2018), and zeta potential was measured across a pH range of 2–12, following Yuan et al. (2011) using a *Malvern Zetasizer Pro Blue* with a reflective index of 2.4 (Marshall et al. 2019).

#### Phytotoxicity test

The seed vigor index (SVI) was determined following the method of Bożym et al. (2021) adapted from standard *NF EN ISO 18763* to evaluate toxicity of various tested solutions using *Lepidium sativum* seed germination. Results were then normalized with respect to control (deionized water). Detailed results without normalization are displayed in Table S2.

#### FTIR-ATR spectroscopy

Surface functional group variations were analyzed using Fourier transform infrared spectroscopy (FT-IR) with a *Thermo Scientific Nicolet IS50 FT-IR spectrophotometer*. Spectra were recorded in the range of 500–4000 cm⁻^1^ for the feedstock, as well as for both pristine and Cu^2+^-loaded biochar. Prior to analysis, biochar samples were finely ground and homogenized with potassium bromide (KBr).

#### Brunauer–Emmett–Teller analysis and porosity

Specific surface area (*S*_*BET*_) of biochar and biomass were measured using an *ASAP 2020 Micromeritics* using N_2_ adsorption at 77 K. The device’s standard error was set at ± 6 m^2^.g^−1^. Prior to measurements, samples were degassed under vacuum at 250 °C. True particle density was determined using a helium displacement pycnometer (*AccuPyc 1330, USA*). Moreover, porosity percentages were calculated according to Chafik et al. (2024).

##### SEM–EDS

Surface and morphology of pristine and Cu^2^⁺-loaded biochars were analyzed using a scanning electron microscope (*SEM Model IT800SHL, JEOL, USA*) with acceleration voltage of 5 kV and 20 kV. Metal distribution and semi-quantitative elemental analysis were performed using energy-dispersive X-ray spectroscopy (EDS) at × 600 magnification and 20 kV. Element weight and atomic percentages were determined through stoichiometry and carbon combination, with data processed with *Aztec®* software.

#### Ultimate analysis

Carbon, hydrogen, nitrogen, oxygen, and sulfur contents were determined using a *Thermo Scientific FLASH 2000 automatic analyzer.* Higher heating value (HHV) and lower heating value (LHV) were calculated following the equation from Frikha et al. (2021). Ion concentrations were determined using a *Thermo Scientific DIONEX AQUION Ion Chromatography System.* Metals contents were analyzed using *ICP-AES (ULTIMA2, HORIBA, San Francisco, USA).*

### Copper Sorption tests, kinetics, and isotherms

#### Sorption tests and sorption isotherms

Cu(II) sorption by biochar was evaluated following the procedure from Chafik et al. (2024). Briefly, a mass of 0.1 g of dried biochar was shaken at 200 rpm for 24 h at room temperature with 10 mL of ultrapure water containing copper (CuCl_2_) at various concentrations (0.1, 0.5, 1.0, 1.5, 2.0, or 2.5 g.L^−1^). After agitation, supernatants were filtered (0.45-µm nitrocellulose filter) and pH and EC were measured. Copper concentrations were recorded by *ICP-AES*. Following the same method, more precise Cu^2+^ sorption by BM was conducted at concentrations of 0.05, 0.1, 0.2, 0.25, 0.3, 0.4, 0.5, 0.75, 1.0, 1.25, 1.5, 2.0, and 2.5 g.L^−1^ at solution pH.

Langmuir and Freundlich sorption isotherm models were used to compare biochar’s Cu^2+^ sorption capacities and were calculated following Chafik et al. 2024 procedure. The Langmuir model assumes monolayer sorption on a homogeneous surface, whereas the Freundlich model is based on multilayer sorption on a heterogeneous surface.

#### Kinetics tests and kinetics isotherms

Cu^2+^ sorption kinetic study was conducted with various contact times with agitation at 200 rpm (5, 10, 15, 30, 45 min and 1, 2, 3,4, 5, 6, 12, 24 h) between 0.1 g of biochar and 10 mL of a 2.0 g.L^−1^ Cu^2+^ solution. After agitation, supernatants were filtered (0.45-µm nitrocellulose filter) and pH and EC were measured. Copper concentrations were recorded by *ICP-AES*. Kinetics pseudo-first-order (PFO), pseudo-second-order (PSO), Elovich and intra-particle diffusion models were determined following Wang and Guo (2020a) procedure. Pseudo-first-order model assumes that sorption is proportional to the difference between the adsorption capacity at equilibrium and the amount adsorbed at a given time, whereas the second first-order model is based on chemical mechanisms. Elovich model indicates a sorption on a heterogeneous surface, while intra-particle diffusion model describes the diffusion of adsorbates inside the pores (Wang and Guo 2020a, 2023; Jin et al. 2021).

### Copper desorption and sorption mechanisms

#### Copper desorption from biochar

Dried biochar was shaken at room temperature at 200 rpm with ultrapure water containing 1.5 g.L^−1^ Cu^2+^ for 24 h to reach equilibrium. Saturated biochar was recovered and dried overnight at 80 °C. Copper desorption was evaluated using various desorbing agents as described in Liu et al. (2024). Briefly, a mass of 0.2 g of Cu-loaded biochar previously prepared was added to 50 mL of either H_2_O, 0.1 M HCl, 0.1 M NaOH, or 0.1M EDTA. Samples were shaken for 6 h at 200 rpm at room temperature. After agitation, supernatants were filtered (0.45-µm nitrocellulose filter) and pH was measured. Copper concentrations were determined by *ICP-AES*. To assess desorption kinetics from BM, various contact times (10, 30, 60, 120, 240, 360, 720, and 1440 min) between desorbing agents and Cu^2+^-loaded biochar were tested using method previously mentioned, with a 2.0 g.L^−1^ solution utilized instead.

#### Quantification of copper sorption mechanism

Contributions of different Cu^2+^ adsorption mechanisms of biochar (ion exchange, complexation, precipitation, and π interaction) were determined following Liu et al. (2024). Briefly, ion exchange (*q*_*exc*_) was determined by measuring K^+^, Na^+^, Ca^2+^, and Mg^2+^ concentration differences between pristine and Cu^2+^-loaded biochars. Complexation (*q*_*com*_) was evaluated by measuring pH shifts before and after Cu^2+^ sorption on acid-washed biochar. Precipitation (*q*_*pre*_) was assessed by comparing Cu^2+^ sorption on pristine and on acid-washed biochar. Finally, π interaction (*q*_*π*_) was determined by the difference.

### Statistical analysis

Data processing was conducted using *Microsoft Excel 2024*. Results are expressed as mean ± standard deviation. Significant differences were determined by ANOVA after verifying normality and homoscedasticity assumptions. Pearson’s correlation and principal component analysis were also performed using R (*v. 4.3.2*).

## Results and discussion

### Feedstock physico-chemical characterization

The feedstock properties are crucial in determining the properties of the resulting biochar. Despite all feedstock biomasses having the same plant origin, they come from different structural components, with either a lignocellulosic or non-lignocellulosic nature, which significantly impacts their composition and transformation during pyrolysis (Ippolito et al. 2020).

#### Proximate analysis

As shown in Table 1, the biomass samples exhibit an acidic pH, ranging from 4.11 ± 0.02 for GM to 5.28 ± 0.06 for P. Electrical conductivity (EC) was high across all samples with the lowest for P (1823.48 ± 92.93 µS.cm^−1^) and the highest for GM (5618.28 ± 324.34 µS.cm^−1^), consistent with previous raw feedstock descriptions (Del Pozo et al. 2022; Anđelini et al. 2023). All feedstocks presented high volatile matter content (> 78%) resulting in limited carbon fixation, below 20%, for all feedstocks in agreement with values reported in the literature (Da Silva et al. 2024; Ferreira et al. 2015; Nunes et al. 2021).

**Table 1:** Physicochemical and compositional properties of the different feedstocks (*GM* grape marc, *P* pruning residues, *GS* grape seeds, *EGS* extracted grape seeds). Results are expressed as mean ± SD. Different letters indicate significant differences among feedstocks for each parameter (*p* < 0.05). Abbreviations: *EC* electrical conductivity, *HHV* higher heating value, *LHV* lower heating value, *SVI* seed vigor index. Oxygen was calculated by difference. “–” indicates values below detection limits

Feedstock	GM		P		GS		EGS	
Proximate								
pH	4.11 ± 0.02	d	5.28 ± 0.06	a	4.73 ± 0.03	c	4.9 ± 0.05	b
EC (µS.cm^−1^)	5618.28 ± 324.34	a	1823.48 ± 92.93	c	2544.04 ± 136.47	b	2368.24 ± 113.01	b
Bulk density (g.cm^−1^)	0.36 ± 0.01	c	0.2 ± 0.0	d	0.62 ± 0.01	a	0.58 ± 0.01	b
Moisture (%)	12.83 ± 3.51	a	8.81 ± 1.71	ab	7.39 ± 0.86	b	10.72 ± 1.10	ab
Volatile matter (%)	79 ± 0.91 b	b	90.04 ± 0.5 a	a	78.51 ± 0.6 b	b	78.64 ± 2.51 b	b
Fixed carbon (%)	16.76 ± 0.91 a	a	7.45 ± 0.5 b	b	18.73 ± 0.6 a	a	18.94 ± 2.51 a	a
Ultimate								
C (%)	45.08 ± 0.38	c	44.36 ± 0.13	d	52.08 ± 0.1	a	50.43 ± 0.31	b
H (%)	5.56 ± 0.06	b	5.61 ± 0.13	b	6.36 ± 0.12	a	6.33 ± 0.12	a
N (%)	2.12 ± 0.03	c	0.84 ± 0.03	d	2.61 ± 0.05	b	3.36 ± 0.06	a
S (%)	0.11 ± 0.01	a	-		0.13 ± 0.01	a	0.14 ± 0.05	a
O (%)	44.91 ± 0.13	b	47.84 ± 0.5	a	36.72 ± 0.63	d	38.12 ± 0.41	c
H/C	1.47 ± 0.00	a	1.51 ± 0.04	a	1.46 ± 0.03	a	1.50 ± 0.03	a
O/C	0.75 ± 0.01	b	0.81 ± 0.01	a	0.53 ± 0.01	d	0.57 ± 0.01	c
HHV (Mj.kg^−1^)	18.89 ± 1.53	c	18.09 ± 0.17	c	20.56 ± 0.08	a	20.1 ± 0.14	b
LHV (Mj.kg^−1^)	16.92 ± 0.15	c	16.81 ± 0.03	c	19.24 ± 0.06	a	18.75 ± 0.12	b
Composition								
Cellulose (%)	14.01 ± 1.73	c	33.14 ± 0.82	a	19.78 ± 0.41	b	15.97 ± 0.34	bc
Hemicellulose (%)	2.87 ± 0.14	c	19.35 ± 5.77	a	10.62 ± 1.62	ab	8.56 ± 2.18	ab
Lignin (%)	29.42 ± 2.39	ab	20.46 ± 5.50	b	31.11 ± 2.82	ab	35.29 ± 2.34	a
Extractable (%)	53.70 ± 0.81	a	27.04 ± 0.55	c	38.48 ± 1.61	b	40.19 ± 0.17	b
Oil (%)	7.09 ± 0.08	c	1.15 ± 0.01	d	16.36 ± 0.09	a	11.39 ± 0.03	b
Ash (%)	4.24 ± 1.94	a	2.51 ± 0.95	b	2.76 ± 0.28	b	2.42 ± 0.03	b
Toxicity								
Normalized SVI (%)	0.00 ± 0.00	b	22.82 ± 6.11	a	0.00 ± 0.00	b	26.91 ± 8.35	a

#### Ultimate analysis

Carbon contents in feedstocks ranged from 44.36 ± 0.13% for P to 52.08 ± 0.1% for GS, while oxygen levels varied between 38.12 ± 0.41% for EGS to 47.84 ± 0.5% for P. Hydrogen content ranged between 5 and 7%, and nitrogen from 0.84 ± 0.03% for P to 3.36 ± 0.06% for EGS. These values are consistent with literature reports, including D’Eusanio et al. (2024) for seed, Marshall et al. (2019) for P, and Demiral and Ayan (2011) for marc. Sulfur content was generally low and undetectable in P, likely due to copper-based treatments (e.g., CuSO₄) being primarily applied to leaves and grapes rather than woody tissues. The elevated oxygen content is associated with surface functional groups, which are expected to degrade during pyrolysis (D’Eusanio et al. 2024).

Higher heating values (HHV) and lower heating values (LHV) ranged from 18 to 20 MJ·kg⁻^1^ and 17 to 19 MJ·kg⁻^1^, respectively, in agreement with previous studies (Sfakiotakis and Vamvuka 2018; Martínez-Gómez et al. 2023; Pardo et al. 2023). GS, with the highest carbon content (> 50%), was most suitable for energy recovery. This was reflected in the highest HHV values observed for GS and EGS (Table 2), which is attributed to the higher lignin content in seed-derived biochars, as lignin has a 30% higher energy content than cellulose or hemicellulose (Pardo et al. 2023).

**Table 2:** Physicochemical properties and elemental composition of biochars derived from different feedstocks (*BM* marc biochar, *BP1* pruning biochar, *BP2* double-pyrolyzed pruning biochar, *BS* seed biochar, *BES* extracted seed biochar). Results are expressed as mean ± SD. Different letters indicate significant differences among biochars for each parameter (*p* < 0.05). Abbreviations: *EC* electrical conductivity, *CEC* cation exchange capacity, *WHC* water-holding capacity, *HHV* higher heating value, *LHV* lower heating value, *SVI* seed vigor index. Oxygen was calculated by difference. “–” indicates values below detection limits

Biochar	BM		BP1		BP2		BS		BES	
Ultimate										
pH	11.74 ± 0.01	a	10 ± 0.02	b	9.75 ± 0.02	c	10.04 ± 0.07	b	9.7 ± 0.02	d
EC (µS.cm^−1^)	15,789.6 ± 243	a	3892.3 ± 230.3	b	2548.5 ± 465	c	1502.8 ± 60.2	e	1650.9 ± 42.7	d
CEC (cmol.kg^−1^)	46.65 ± 1.84	a	36.98 ± 0.77	b	35.27 ± 2.67	b	20.7 ± 3	c	21.77 ± 0.96	c
WHC (%)	164.85 ± 6.32	d	386.48 ± 11.18	a	357.56 ± 7.47	b	196.82 ± 3.6	c	206.39 ± 6.85	c
Ash (%)	27.49 ± 0.71	a	11.25 ± 0.29	b	7.45 ± 0.4	d	9.02 ± 0.79	c	9.54 ± 0.11	c
S_BET_ (m^2^.g^−1^)	55		185		253		79		214	
Moisture (%)	27.5 ± 0.08	a	2.47 ± 0.01	e	7.2 ± 0.13	b	6.22 ± 0.08	c	5.72 ± 0.08	d
Bulk density (g. cm^−1^)	0.47 ± 0.03	a	0.21 ± 0.02	c	0.21 ± 0.02	c	0.34 ± 0.01	b	0.35 ± 0.04	b
Real density (g. cm^−1^)	1.84		1.69		1.75		1.69		1.84	
Porosity (%)	74.42		87.56		88.03		79.83		80.97	
Volatile matter (%)	23.64 ± 0.22	a	24.53 ± 0.77	a	22.06 ± 1.6	a	17 ± 0.4	b	16.07 ± 1.24	b
Fixed carbon (%)	48.46 ± 0.22	d	64.22 ± 0.77	c	70.49 ± 1.6	b	73.98 ± 0.4	a	74.39 ± 1.24	a
Proximate										
C (%)	63.38 ± 1.5	c	77.51 ± 0.62	b	80.58 ± 0.9	a	81.98 ± 3.96	a	73.18 ± 6.25	b
H (%)	1.28 ± 0.09	bc	1.9 ± 0.11	a	1.97 ± 0.27	a	1.63 ± 0.13	ab	1.1 ± 0.08	c
N (%)	1.41 ± 0.05	a	0.86 ± 0.03	bc	0.68 ± 0.03	c	1.23 ± 0.18	a	1.16 ± 0.19	ab
S (%)	0.09 ± 0.05		-		-		-		-	
O (%)	32.63 ± 1.47	a	18.63 ± 0.88	bc	15.53 ± 1	bc	13.74 ± 3.99	c	22.86 ± 5.94	b
H/C	0.24 ± 0.02	a	0.29 ± 0.01	a	0.29 ± 0.01	a	0.24 ± 0.03	ab	0.18 ± 0.01	b
O/C	0.39 ± 0.03	a	0.18 ± 0.01	b	0.14 ± 0.01	b	0.13 ± 0.04	b	0.24 ± 0.08	b
HHV (Mj.kg^−1^)	20.45 ± 0.43	c	24.86 ± 0.25	ab	25.78 ± 0.42	a	25.92 ± 1.09	a	23.08 ± 1.83	bc
LHV (Mj.kg^−1^)	20.17 ± 0.43	c	24.46 ± 0.22	ab	25.36 ± 0.37	ab	25.58 ± 1.09	a	22.85 ± 1.81	bc
Toxicity										
Normalized SVI (%)	4.19 ± 3.63	c	87.39 ± 26.96	ab	71.31 ± 23.15	b	79.78 ± 18.04	b	92.29 ± 6.38	a

#### Structural composition

GM exhibited a high content of extractable compounds (53.70 ± 0.81%) but relatively low levels of hemicellulose (2.87 ± 0.14%) and cellulose (14.01 ± 1.73%), consistent with previous reports (Li et al. 2016; Frikha et al. 2021). In contrast, P contained approximately half the extractables of GM (27.04 ± 0.55%) and showed higher hemicellulose (19.35 ± 5.77%) and cellulose (33.15 ± 0.82%) contents, in agreement with findings by Videgain et al. (2021). GS and EGS displayed similar compositions and the highest lignin contents among the feedstocks, with values of 31.11 ± 2.82% and 35.29 ± 2.34%, respectively, supporting the observation that grape seeds are among the most lignin-rich plant tissues (Yedro et al. 2015). High lignin content promoted char formation during pyrolysis, while cellulose and hemicellulose contribute to the formation of oxygen-containing functional groups. Lignin decomposes at higher temperatures, producing phenolic radicals that re-polymerize into solid char, whereas cellulose and hemicellulose decompose more easily due to their simpler structures, yielding to a broader range of oxygenated functional groups (Da Silva et al. 2024; Hassan et al. 2020). Ash content was highest in GM (4.24 ± 1.94%), while the other feedstocks showed lower values (~ 2.5%). GS had the highest oil content (16.35 ± 0.09%), consistent with values reported by Jimenez-Cordero et al. (2013), whereas S exhibited the lowest oil content (1.15 ± 0.01%), as expected for woody biomass.

### Feedstock thermogravimetric analysis (TGA/DTG)

Thermogravimetric analysis (TGA) and derivative thermogravimetric (DTG) profiles for the studied feedstocks are shown in Fig. S1. Four major peaks were observed between room temperature and 600 °C, corresponding to water loss and the thermal degradation of hemicellulose, cellulose, and lignin—the primary organic constituents of plant-based biomass (Xu et al. 2013). Variations in the TGA and DTG curves among the feedstocks reflect the differing compositions in hemicellulose, cellulose, and lignin (Table 1). The initial mass loss in the DTG curves (0–120 °C) is attributed to moisture evaporation, followed by the release of bound and structural water, as well as the removal of semi-volatile compounds either formed during pyrolysis or initially present (120–250 °C) (D’Eusanio et al. 2024). The earlier occurrence of the first DTG peak in P (Fig. S1.B) suggests a higher proportion of free water that is readily released, likely due to the vascular structure characteristic of this woody biomass. Indeed, the dehydration processes during pyrolysis lead to the formation of pores on the biochar surface (Meng et al. 2025), suggesting higher porosity on future biochar made from pruning.

The second peak, occurring between 250 and 310 °C, is associated with the decomposition of hemicellulose, while the third peak, observed around 360 °C, corresponds to cellulose degradation (Xu et al. 2013; Boundzanga et al. 2022). This third peak results in the greatest mass loss for P (Fig. S1.B), consistent with its high cellulose content (33.1%) among the studied feedstocks. Lignin decomposition occurs over a broad temperature range (Xu et al. 2013), with significant decomposition typically observed from 375 to 600 °C (Videgain-Marco et al. 2021; Boundzanga et al. 2022). However, net lignin decomposition peaks were not observed likely due to the overlapping degradation of fatty acids, which occurs around 400 °C (D’Eusanio et al. 2024). This was particularly notable in GS, EGS, and GM (Fig. S1. A, C, D), all of which have significant oil content (Table 1). These feedstocks exhibited a pronounced DTG peak and substantial mass loss in this temperature range, in contrast to P (Fig. S1.B), which showed minimal mass loss in this region.

### Differential scanning calorimetry (DSC/DTG)

Differential scanning calorimetry (DSC) and derivative thermogravimetric (DTG) results are presented in Fig. S2 for the studied feedstock. In the DSC curves, regions where the signal is greater than 0 µV/mg indicate an endothermic phenomenon, while regions where the signal is less than 0 µV/mg correspond to an exothermic process. The endothermic peak observed between 100 and 150 °C is attributed to the energy consumption associated with water evaporation (Bryś et al. 2016). Conversely, the prominent exothermic peak observed between 250 and 450 °C for the studied feedstocks corresponds to energy release due to carbonization and the thermal decomposition of hemicellulose, cellulose, and lignin during the pyrolysis process (Chen et al. 2014; Shen et al. 2015; Bryś et al. 2016). Moreover, a minor exothermic shoulder at 400 °C observed in all samples suggests the final stage of lignin degradation at this temperature, which also consumes energy (Cagnon and Py 2012).

### Physicochemical characterization of biochars

In this study, five biochars produced from above-characterized viticultural residues were analyzed. As shown in Table S3, all the biochar analyzed showed similar properties as those described in literature.

#### Proximate analysis

Compared with their respective feedstocks, pyrolysis increased pH, resulting in a shift toward alkaline conditions (Table 2). BM exhibited the highest pH (11.74 ± 0.01), which is commonly attributed to the volatilization of acidic organic compounds, the relative enrichment of alkaline mineral ash, and changes in surface chemistry induced by pyrolysis (Gaskin et al. 2008; Lee et al. 2017). Indeed, BM showed a notably high ash content of 27.49 ± 0.71%, as confirmed by ion concentrations in Table S1, followed by BP1 (11.25 ± 0.29%), which confirmed a known positive correlation between ash content and pH (Jin et al. 2016). Ash contents were also closely related to electrical conductivity (EC); BM exhibited an EC of 15,789.6 ± 243 µS·cm⁻^1^, approximately 10 and 5 times higher than seed-derived biochars (BS, BES) and pruning-derived biochars (BP1, BP2), respectively. High biochar pH and EC favored metal sorption by enhancing precipitation and complexation of heavy metals (Thomas et al. 2020; Liang et al. 2021), while the elevated EC, driven by ash and ionic content (P, Ca, K), may promote co-precipitation of heavy metals (Nguyen et al. 2023; Guo et al. 2024).

Regarding cation exchange capacity (CEC), BM again had the highest value (46.65 ± 1.84 cmol·kg⁻^1^), followed by BP1 (36.98 ± 0.77 cmol·kg⁻^1^), highlighting their stronger potential for metal sorption (Liang et al. 2021; Lima et al. 2022). Fixed carbon content, calculated by considering moisture, ash, and volatile matter, was lowest for BM, likely due to its high ash content, elevated moisture (27.5 ± 0.08%), and volatile matter (23.64 ± 0.22%). In contrast, seed-derived biochars (BS, BES) showed the highest fixed carbon, correlating with their lower volatile matter content (< 17%).

Water holding capacities (WHC) varied from 164.85 ± 6.32% for BM to 357.56 ± 7.47% and 386.48 ± 11.18% for pruning-derived biochars, reflecting differences in porosity. BM had the lowest porosity (74.42%), while BP2 showed the highest (88.03%), consistent with the positive relationship between porosity and WHC reported by Alotaibi and Schoenau (2019) and Batista et al. (2018). A similar trend was observed for specific surface area (*S*_*BET*_), which is closely linked to porosity, as highly porous structures typically exhibited greater surface areas (Shinogi and Kanri 2003). BP2 displayed the highest S_*BET*_ (253 m^2^.g^−1^), whereas BM had the lowest (55 m^2^.g^−1^). Interestingly, BES showed a S_*BET*_ of 214 m^2^.g^−1^, possibly due to polyphenol extraction before processing. Additionally, the greater porosity observed in wood-derived biochar compared to seed-derived biochar was likely linked to the presence of conductive vascular tissues in woody biomass (Chafik et al. 2024). Both porosity and *S*_*BET*_ were identified as key factors facilitating metal sorption (Li et al. 2017; Thomas et al. 2020; Meng et al. 2025). While *S*_*BET*_ correlated with WHC in some cases (e.g., BM and BS), this relationship weakened at higher *S*_*BET*_ values. For example, BP1 had higher WHC than BES, despite lower *S*_*BET*_. This suggested that pores in BES were not fully accessible to water. This difference might also be explained from the higher cellulose and hemicellulose levels among pruning. Upon degradation, these components produced hydrophilic oxygen-containing functional groups. Conversely, seed biochars contained higher lignin contents, resulting in higher biochar yields but fewer hydrophilic functional groups (Hassan et al. 2020).

Water retention profile (% mass) (Fig. S3) revealed that all biochars improved soil moisture retention at each suction force. As expected, the biochars with the highest WHC and porosity exhibit the best water retention (Liu et al. 2017), particularly pruning biochar (BP1, BP2) followed by marc biochar (BM) and then seed biochars (BES, BS). Pruning biochar notably enhanced water retention consistent with findings from Marshall et al. (2019). In contrast, non-woody seed biochars showed no significant difference from the control soil in most cases. These results suggest that pruning’s biochar could therefore be beneficial for improving agricultural soils, particularly sandy soils (Wei et al. 2023), although a study extending to the permanent wilting point (−1500 kPa) is necessary.

#### Ultimate analysis

As expected for biochar, carbon (C) was the most abundant element in all tested samples, ranging from 81.98 ± 3.96% (BS) to 63.38 ± 1.5% (BM), followed by oxygen (O) with contents between 32.63 ± 1.47% (BM) and 13.74 ± 3.99% (BS). Hydrogen (H) and nitrogen (N) contents remained below 1.9%, while sulfur (S) was only detected in BM at 0.09% (Table 2). The elemental composition of biochar largely reflected the chemical structure of the feedstocks (Meng et al. 2025). Oxygen was primarily associated with functional groups, while hydrogen was linked to aliphatic and aromatic structures. Both were present in lower proportions than carbon, probably due to volatilization during pyrolysis. Carbon tended to condense into stable aromatic structures, while oxygen and hydrogen were released—via the decomposition of functional groups such as hydroxyl and carboxyl groups—as water (Chen et al. 2019; Ippolito et al. 2020; De Souza Souza et al. 2021). The remaining oxygen-containing functional groups may interact with heavy metals through ion exchange or complexation (Meng et al. 2025). In addition, the stable carbon structure of biochar enhanced its resistance to degradation and provided π-electron-rich sites for metal cation–π interactions (Meng et al. 2025).

The atomic H/C and O/C ratios provided insights into the structural transformations occurring during the carbonization. In general terms, these ratios decreased with increasing pyrolysis temperatures (Spokas 2010; Ippolito et al. 2020) and served as key indicators of biochar surface properties. In the literature, the H/C ratio reflected the degree of aromaticity (lower H/C = higher aromaticity), whereas the O/C ratio indicated both polarity (higher O/C = higher polarity) and aromaticity (lower O/C = higher aromaticity) (Chafik et al. 2024). The obtained biochars exhibited low H/C ratios (0.18–0.29), suggesting high aromaticity levels (< 0.7) and long-term stability in soil. O/C ratios were below 0.2 for all samples except BM (0.39), suggesting, for the latter, moderate oxidative stability (Spokas 2010). As mentioned above, the limited oxygenated functional groups made most of biochars characterized by low polarity and hydrophobicity. The higher O/C ratio for BM suggests a greater presence of oxygenated functional groups, which may potentially enhance BM metal sorption capacity (Thomas et al. 2020). Plotting H/C versus O/C ratio on a Van Krevelen diagram (Fig. S4) for both biochar and feedstock allowed a better understanding of the carbonization process during pyrolysis. The decrease in the H/C and O/C ratios after pyrolysis was typical of carbonization (Tag et al. 2016), during which OH-containing volatile elements (e.g., acetic acid, methanol, water) were eliminated by dehydration and decarbonylation reactions, which leaving behind a more stable carbonaceous residue comparable to charcoal (Wyn et al. 2020).

Ion content analysis (Table S1) revealed that BM contained significant levels of nutrients (e.g., Ca^2^⁺, Mg^2^⁺, NO₂⁻), consistent with its origin from grape marc, a nutrient-rich by-product of the wine industry (Kurćubić et al. 2024), and containing negligible concentrations of trace elements, below regulatory thresholds in all cases (*EBC and IBI standards*) making it suitable as fertilizer (Zabaniotou et al. 2018). This results emphasize the previously described marc-derived biochar potential in fertilization (Manolikaki et al. 2016; Ibn Ferjani et al. 2019). Additionally, BM exhibited the highest PO₄^3^⁻ content among the tested biochars (4812.2 ± 246.3 mg·kg⁻^1^), over ten times higher than the rest of biochars tested, and up to 4% (w/w) in exchangeable K⁺. Its elevated levels of exchangeable cations may also contribute to metal precipitation (Xu et al. 2013; Nguyen et al. 2023), likely contributed to its sorption capacity.

As expected, the higher heating value (HHV) and lower heating value (LHV) were higher for biochars with elevated carbon and low oxygen contents (BP2 and BS), and lower for BM which had the lowest carbon and highest oxygen contents. These values increased by up to 25% compared to feedstocks, highlighting the benefits of pyrolysis for energy production, energy densification, and enhanced resistance to biological degradation (Nunes et al. 2021).

### FT-IR spectra analysis

**Fig. 1:**
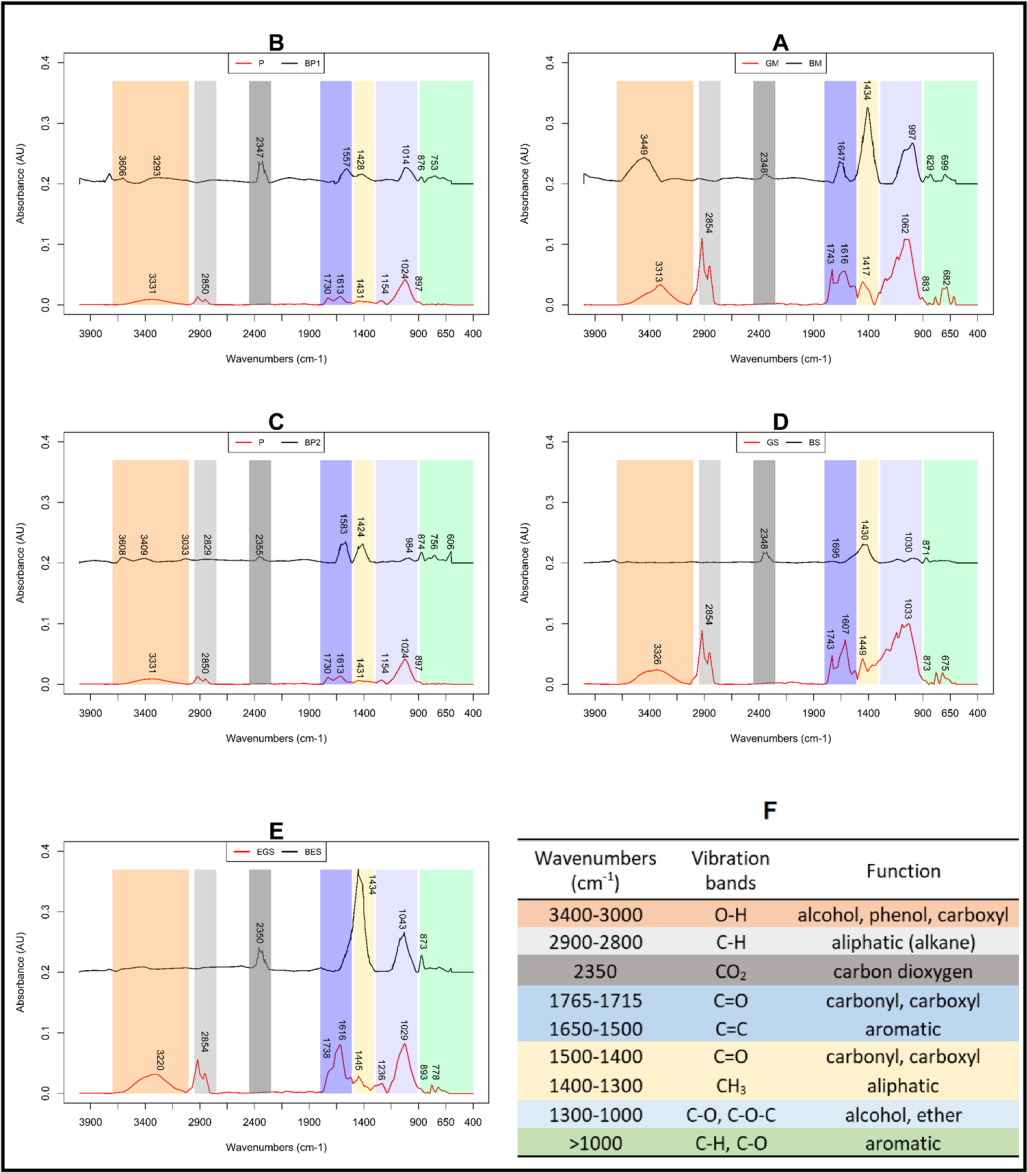
FT-IR spectra of five biochars and their corresponding feedstocks: **A** GM and BM; **B** P and BP1; **C** P and BP2; **D** GS and BS; **E** EGS and BES. Spectra show absorbance (A.U.) versus wavenumber (cm^−1^), with key peaks labeled. Vibrational bands and their corresponding functional groups are summarized in panel (**F**). Spectral regions were region 1 (3000–3400 cm^−1^; orange), region 2 (2900–2800 cm^−1^; light grey), region 3 (2350 cm^−1^; dark grey), region 4 (1765–1500 cm^−1^; dark blue), region 5 (1500–1300 cm^−1^; yellow), region 6 (1300–1000 cm^−1^; light blue), and region 7 (< 1000 cm^−^.^1^; green)

FT-IR spectra (Fig. 1) were analyzed by comparing the spectral regions (1) biochar and feedstock with each other and (2) biochar with its corresponding biomass. Key spectral regions are color-coded for easier interpretation, corresponding to the information in Fig. 1F.

#### Feedstock FT-IR spectra analysis

The surface functional groups present within biomasses are mainly associated with hemicelluloses, cellulose, and lignin structures, and relative proportions are primarily discussed. First, absorption between 3000 and 3400 cm⁻^1^ arises from OH bonds in carboxylic acids, alcohols, and phenols in the biomass (Da Silva et al. 2024), and is pronounced for GM, GS, and EGS (Fig. 1A, D, E) but less developed for P (Fig. 1B, C), indicating that prunings contain fewer hydroxyl groups. The absorption between 2900 and 2800 cm⁻^1^ originates from CH bonds in aromatic, alkyl, and aliphatic groups within biomass structures (Da Silva et al. 2024) and in residual oils (Missaoui et al. 2017). This region showed higher intensity for GM, GS, and EGS (Fig. 1A, D, E) than for P (Fig. 1B, C), consistent with the higher oil content in marc and seeds compared with the woody parts (Table 1).

The peaks between 1765 and 1500 cm⁻^1^ were associated withs carbonyl groups (C = O), indicating the presence of polysaccharides such as hemicelluloses (Da Silva et al. 2024), aromatic rings (C = C) from lignin (Calderón et al. 2006; Da Silva et al. 2024), and methoxyl (O-CH_3_) characteristic groups of lignin (Yang et al. 2007; Xu et al. 2013). These peaks were higher in GS and EGS (Fig. 1D, E) due to their higher lignin contents (Table 1). Absorption between 1500 and 1300 cm⁻^1^ results mainly from aliphatic chains (CH₂, CH₃) and carbonyls (C = O) around 1430 cm^−1^ typical of saccharides and lignin found in biomass (Sánchez Orozco et al. 2014). Absorption between 1300 and 1000 cm⁻^1^ corresponds to C–O–C structures and alcohol-OH groups, typical of oxygenated functional groups found in cellulose (Xu et al. 2013). Additionally, the band near 1000 cm⁻^1^ for all feedstocks is attributed to guaiacyl-type lignin structures (Sun and Tomkinson 2002) and saccharide-like structures, such as hemicelluloses and cellulose (Missaoui et al. 2017). Finally, absorption peaks below 1000 cm⁻^1^ reveal the presence of aromatic constituents, particularly for GM, GS, and EGS (Fig. 1A, D, E). Bands around 890 cm⁻^1^ reveal presence of C–C, the carbon skeleton of cellulose (Calderón et al. 2006) for all biochar.

Overall, the FT-IR spectra of the feedstocks show minimal variation, with distinct peaks clearly corresponding to the major constituents of plant biomass, namely hemicelluloses, cellulose, and lignin. However, the majority of the peaks were less developed in P than in the other three biomasses mainly due to the relatively different lignin and oil compositions.

#### Changes during pyrolysis

During pyrolysis, feedstock functional groups vanish or are transformed. These include the C = O stretch specific to hemicelluloses (1765-1715cm⁻^1^) and aliphatic chains (2900–2800 cm⁻^1^) fades for all biochar. Additionally, the OH band in the first spectral region (3400–3000 cm^−1^) fades with pyrolysis for BS and BES (Fig. 1D, E). These losses are consistent with the fact that pyrolysis degrades oxygenated and hydrogen-rich compounds (Yuan et al. 2011; Tomczyk et al. 2020). However, certain functional groups remain intact, such as the OH vibration band between 3000 and 3400 cm⁻^1^ for BM (Fig. 1A) and the band around 1430–1450 cm⁻^1^ for all biochar, and especially BES (Fig. 1E) which corresponds to carbonyl (C = O). The band around 890 cm⁻^1^ typical from cellulose is no longer present in BM but persists in the rest of biochars. Oxygen containing functional groups (hydroxyl and carboxyl) were key groups for metal complexation (Lin et al. 2025). After pyrolysis, a band at 2350 cm⁻^1^ was identified in all biochars in the second spectral region (Fig. 1, dark grey); the new band could be attributed to sequestrated CO₂ within the porous structure (Schott et al. 2021). In addition, aromatic compounds are formed (> 1000 cm^−1^) in all biochar but specially in in BP1 and BP2 (Fig. 1B, C). These aromatics compounds, which are well-known to be produced during pyrolysis (Keiluweit et al. 2010; Al-Wabel et al. 2013), play an important role in cation-π interactions for sorption (Li et al. 2017).

Moreover, some of the newly observed bands in BS and BES could possibly be associated with PO₄^3^⁻, bands around 1040 cm⁻^1^ (Fig. 1D, E), and for all biochars, a new band at around 1415 cm⁻^1^ could be related with CO₃^2^⁻, which suggests that these biochars might precipitate Cu by producing copper phosphates or copper carbonates (Zhang et al. 2021). Bands around 1030 cm⁻^1^ may potentially reflect the presence of Si in seed biochar, indicating that copper silicate formation may occur (Yuan et al. 2011). The presence of such minerals in biochar is further supported by the data in Table S1 and the EDS spectrum (Table S9 and S10). Unlike biomass, where little differences were observed among feedstocks, biochars exhibit multiple variations depending on the raw material. These variations can be explained by two factors: (1) the difference in hemicelluloses/cellulose/lignin proportion in the different feedstocks and their response to pyrolytic temperatures (Mukome et al. 2013) and (2) variation in pyrolysis conditions, in particular, the final heat temperature (Açıkalın et al. 2012).

#### FT-IR spectra of copper-loaded biochar

To assess the copper (Cu) sorption mechanisms, biochars before and after copper sorption were analyzed using FT-IR. Physicochemical interactions occurring during the sorption process may lead to structural modifications of the adsorbent, which can be observable through FT-IR analysis (Batool et al. 2017).

A comparison of spectra before and after Cu sorption reveals notable differences, highlighting that sorption mechanisms indeed take place on the surface of the biochar. After copper sorption, significant shifts are observed in the region attributed to hydroxyl groups (OH 3000–3700 cm^−1^) and carbonyl-carboxyl groups (i.e., C = O 1400–1500 cm^−1^), emphasizing the role of these functional groups in the sorption of Cu^2+^ by complexation as reported by several authors (Tong et al. 2011; Mahdi et al. 2018; Zhang et al. 2021)*.* More precisely, shifts were, in cm^−1^, 3349 to 3451 cm^−1^ for BM; 3606 to 3634 cm^−1^ for BP1; 3409 to 3214 cm^−1^ for BP2 and 1434 to 1426 cm^−1^ for BM; 1424 to 1408 cm^−1^ for BP2; 1430 to 1421 cm^−1^ for BS; and 1434 to 1435 cm^−1^ for BES. In addition, other shifts occur after sorption for all biochars, such as in the regions < 1000 cm^−1^ (in cm^−1^, 829 to 848 for BM; 876 to 880 and 753 to 751 cm^−1^ for BP1; 874 to 832 cm^−1^ for BP2; 871 to 873 cm^−1^ for BS; and 873 to 876 cm^−1^ for BES), which mainly corresponds to aromatic compounds that may include carboxyl functional groups (Yuan et al. 2011; Tong et al. 2011). Changes in aromatic structure were also visible for BP1 and BP2 around 1600 cm^−1^ (in cm^−1^, 1557 to 1575 for BP1 and 1583 to 1643 for BP2) and corresponding to the C = C vibration band, indicating that Cu^2+^-π interaction might contribute to Cu sorption on the biochar surface (Zhang et al. 2021; Liu et al. 2024). Moreover, intense peaks newly formed around 3300 cm^−1^ for BM may be assigned to the formation of Cu(OH)_2_ (Batool et al. 2017).

### Copper sorption capacity of biochars

**Fig. 2:**
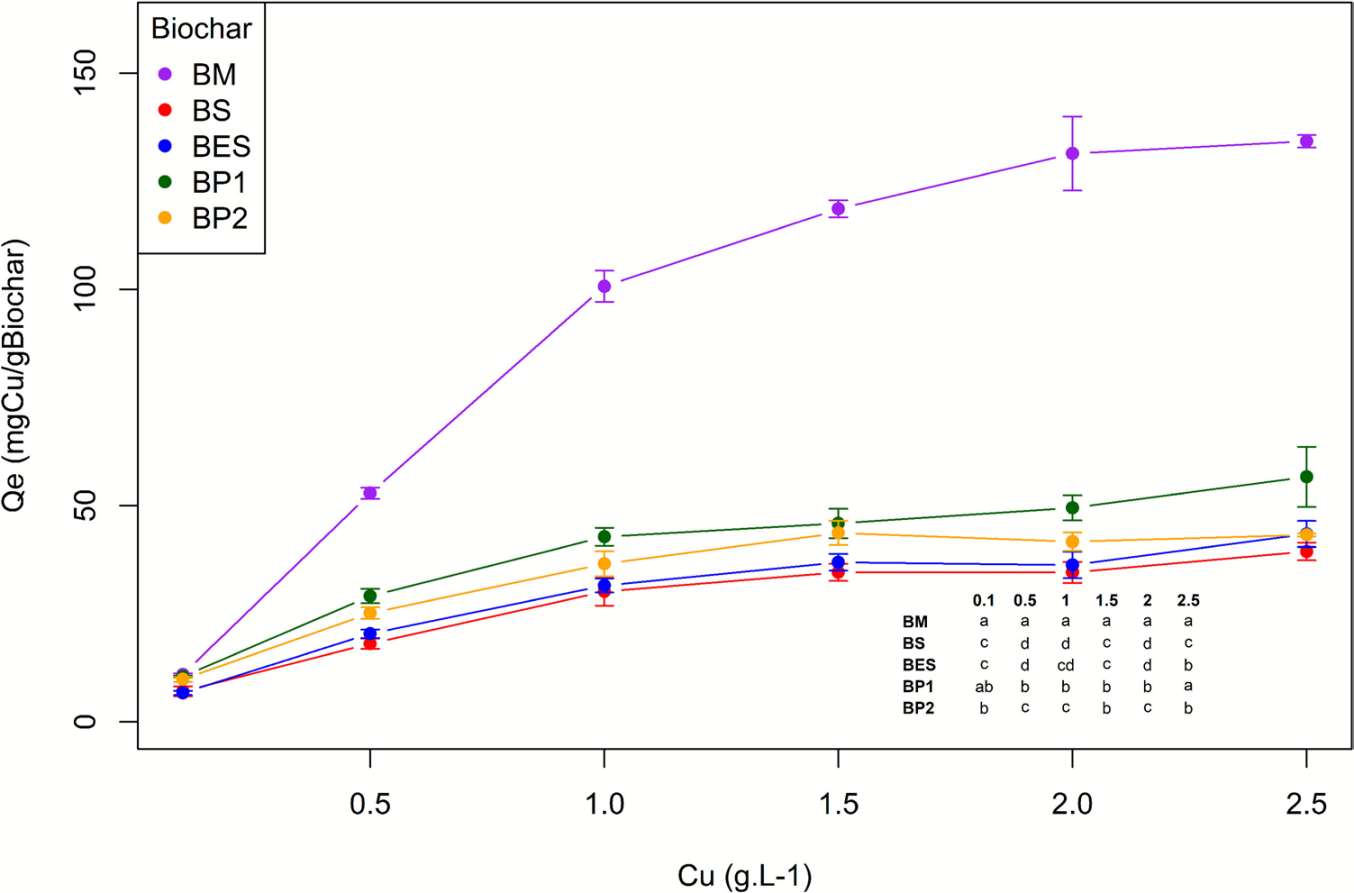
Sorption capacities of various biochars (BM, marc biochar; BS, seed biochar; BES, biochar extracted seed; BP1, pruning biochar; BP2, pruning biochar with two pyrolysis). Statistical comparisons showing significant differences between biochars at the same metal concentration are shown in the table on the lower right corner of the graph (*p* < 0.05; *n* = 5 ± SD)

As shown in Fig. 2, Cu sorption capacity peaked at 2.5 g. L^−1^ for the five biochar, with *Q*_*e*_ (SorbedCu.g_Biochar_^−1^) of 134.26 ± 1.44, 56.60 ± 6.98, 43.18 ± 0.33, 39.35 ± 2.03, and 43.38 ± 3.01 for BM, BP1, BP2, BS, and BES, respectively. Seed-derived biochars had the lowest maximal Cu sorption, approximately 30% lower than pruning biochar (BP1), which was himself more than two times smaller than marc biochar sorption capacity. During the sorption test, pH gradually decreased while EC gradually increased with increasing Cu doses, reaching a solution pH of approximately 5.6 and an EC of 7500 µS·cm⁻^1^ at the highest Cu concentration, with only slight differences among the biochars.

**Fig. 3:**
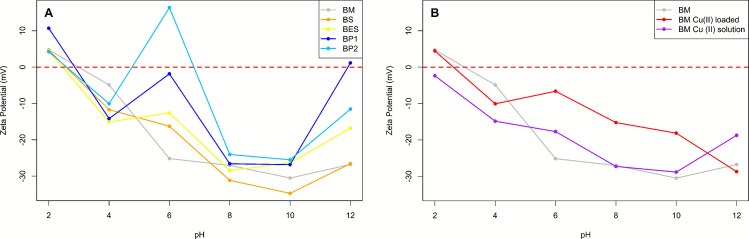
**A** Zeta potential variations of biochars as a function of pH. **B** Zeta potential of BM biochar before and after Cu^2+^ loading, and in presence of 5 mg L^−1^ Cu solution, across pH range. Dashed red line marks zero potential

These results were explained by the preference of copper to be sorbed by complexation on O-rich surface functional group along with electrostatic interactions (Meng et al. 2025) which are numerous on the biochar of marc as supported by FT-IR results (Fig. 1), oxygen content by EDS (Table S6 and S7), and elemental analysis (Table 2) compared to the other biochar. Additionally, Da Silva et al.’s (2024) study mentioned that complexation and electrostatic interaction played an important role in the Cu^2+^ adsorption using a marc biochar with a *Q*_*max*_ below our value (42 mg. g^−1^). This highlights the importance of zeta potential and, in particular the iso-electric point (Fig. 3) which shows that biochar is negatively charged at solution pH (iso-electric point < 3), favoring electrostatic attraction of Cu^2+^. Nonetheless, the good sorption capacities of marc biochar are not linked to its porosity or its *S*_*BET*_ because these parameters are below the values of the other biochar considered (Table 2), consistent with other studies (Mei et al. 2025). The superior performance of BP1 and BP2 compared to BS could be explained by their larger BET surface area of approximately 75% allowing a higher exchange surface as well as a CEC 1.75 times higher and a higher exchangeable cation content (2 times more Mg^2+^ and 4 times more K^+^; Table S1).

Moreover, marc and seed biochars (BS and BES) fit Langmuir sorption isotherm (R^2^ > 0.88; Table S4), indicating a Cu^2+^ chemical adsorption on a monolayer and a homogeneous adsorption distribution on biochar surface (Wang and Guo 2020b), as reported by many authors concerning Cu^2+^ sorption (Araya et al. 2021; Cao et al. 2019; Jin et al. 2021; Tong et al. 2011). Conversely, pruning biochar (BP1 and BP2) fitted the Freundlich isotherm (R^2^ > 0.9) suggesting that sorption processes involved a heterogeneous distribution of adsorption sites on biochar’s surface and that Cu^2+^ adsorption occurred on multilayer surfaces (Wang and Guo 2020b) as reported also by many authors (Bandara et al. 2020; Zhang et al. 2021; Da Silva et al. 2024). The *n*-values were greater than 1 (Table S4), indicating that the adsorption of Cu^2+^ onto the adsorbents was favorable under the studied experimental conditions, while a K_L_ between 0 and 1 implies an easy adsorption process (Cao et al. 2019; Guo et al. 2025).

Due to its unique physical properties and higher Cu sorption, the BM sorption behavior was further characterized (Fig. S5A), confirming a chemisorption-driven process consistent with the Langmuir isotherm (R^2^ 0.83). The sorption curve displayed an initial linear phase up to 1.0 g·L⁻^1^ Cu, followed by a plateau with minimal increase in sorption up to 2.5 g·L⁻^1^. A *Q*_*max*_ of 130.73 ± 6.1 mg_Cu_·g_BM_⁻^1^ was reached at 2.5 g·L⁻^1^, in agreement with Fig. 2. Although the Cu sorption capacity of BM lies in the upper range of reported values, multiple studies have reported similar *Q*_*max*_ values in the range of 100 mg g⁻^1^ Cu adsorption. For instance, biochar produced from spent coffee grounds at 800 °C, seaweed-derived biochar prepared at 500 °C, or kelp-based biochar produced at 600 °C exhibited *Q*_*max*_ of 112.3 mg g⁻^1^, 131.58 mg g⁻^1^, and 93.55 mg g⁻^1^, respectively (Katiyar et al. 2021; Araya et al. 2021; Hu et al. 2025).

Finally, Fig. S5B presents the Cu sorption kinetics on BM; sorption is already significant after 5 min (*Q*_*e*_ 38.08 ± 9.96 mg.g^−1^), and then, between 5 and 60 min, 85% of the total sorption occurred (*Q*_*e*_ 98.04 ± 5.04 mg.g^−1^), followed by a slower uptake until 1440 min, when the near-equilibrium was reached (*Q*_*e*_ 116.26 ± 3.77 mg.g^−1^). During the same period, the solution pH increased by 11% (Table S8). These Cu sorption kinetics were fitted well in the pseudo-second-order (PSO) models (R^2^ = 0.85), indicating chemisorption on a heterogeneous surface (Wang and Guo 2020a), which, in addition, is consistent with the sorption mechanisms identified for BM (Fig. 5). These findings align with Da Silva et al. (2024), who reported equilibrium at 60 min and PSO as the best fit for grape marc biochar, and Zhang et al. (2021), who found similar trends with cow manure biochar for Cu sorption.

### Zeta potential and surface charge behavior

The zeta potential (ZP) measurements revealed the pH-dependent surface charge behavior of biochars. Biochar surface contains various functional groups (carboxyl, phenolic, hydroxyl, etc.) that can protonate at low pH or deprotonate at high pH, altering the surface charge (Hunter 1982). As pH increases, deprotonation increases the negative charge, favoring electrostatic attraction between biochar and Cu^2^⁺, thereby promoting sorption. The isoelectric point (IEP, or pH_pzc_) is the pH at which biochar’s surface net charge is neutral (Mukherjee et al. 2011). At pH values above the IEP, the biochar surface becomes more negatively charged, attracting positively charged ions (Peqini et al. 2025), increasing Cu^2+^ adsorption (Jin et al. 2021).

Zeta potential measurements as a function of pH (Fig. 3A) show that BM, BS, and BES decrease from ZP > 0 mV at pH 2 to ZP < −25 mV at pH 10 with isoelectric points (IEP) around pH 3, and a slight ZP increase at pH 12. In contrast, BP1 and BP2 exhibit a wavelike trend, with BP2 reaching a positive ZP at pH 6 indicating an IEP of 6.80. At most pH values, biochar maintains a negative ZP, which becomes more pronounced between pH 8–10, enhancing cation reactivity (Meier et al. 2017; Yuan et al. 2011). The ZP decrease with increasing pH may be attributed to the deprotonation of carboxyl and hydroxyl functional groups, highlighting the presence of oxygen-containing functional groups (Song et al. 2019) on biochar surface as already seen by FT-IR (Fig. 1) Moreover, biochar IEPs range from 2 to 7, indicating that Cu^2+^ interaction via electrostatic attraction is favored at pH values above the IEP (Jin et al. 2021). Variations in ZP-pH curves suggest differences in functional groups composition, dependent on feedstock type, as supported by FT-IR spectra (Fig. 1). Electrostatic ion adsorption does not significantly affect colloidal surface charge, as the ions are not chemically bound. However, specific ion adsorption such as Cu^2+^ complexation with biochar functional groups modifies ZP due to chemical bonding (Tong et al. 2011).

To assess Cu^2+^ sorption mechanism, ZP was measured for BM, BM Cu^2+^-loaded, and BM in a 5 mg.L^−1^ Cu^2+^ solution (Fig. 3B). All exhibited a similar ZP decline with increasing pH, with an isoelectric point (IEP) around 3. At pH 10, ZP values reached −30 mV for BM and −18 mV for BM Cu^2+^-loaded and −28 mV for BM in Cu solution, indicating differences in surface properties due to sorption or Cu presence. Sorption shifts the ZP-pH positively (Tong et al. 2011; Xu et al. 2011; Jia et al. 2013), suggesting Cu^2+^ interaction with negatively charged surface functional groups on biochar (Tong et al. 2011), thereby reducing surface negativity. The greatest difference between pristine and Cu^2+^-loaded biochar occurs at pH 5–6, where Cu sorption peaks due to functional groups’ deprotonation that increases negative charges for Cu^2+^ binding (Tong et al. 2011). Cu^2+^ remains soluble at this pH but forms Cu(OH)_2_ at higher pH (Jin et al. 2021; Zhang et al. 2021). Ultimately, BM in 5 mg.L^−1^ Cu^2+^ solution displays a more negative ZP than BM Cu^2+^-loaded but remains closer to pristine BM, suggesting dissolved Cu^2+^ partially influences surface charge. Differences between BM in Cu solution and BM Cu^2+^-loaded may result from a balance between Cu^2+^ adsorption and ions distribution in solution (Tong et al. 2011).

### SEM–EDS

#### Pristine biochar

**Fig. 4:**
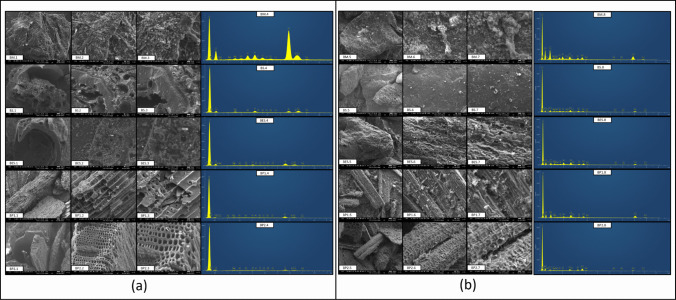
SEM images and corresponding EDS spectra of pristine (**a** 1–4) and Cu-loaded (**b** 5–8) biochars (BM, marc biochar; BS, seed biochar; BES, biochar extracted seed; BP1, pruning biochar; BP2, pruning biochar with two pyrolysis). SEM images taken at magnifications: × 60 (1/5), × 300 (2/6), and × 600 (3/7); EDS spectra were shown in 4/8

Differences in the surface morphology and structural characteristics of each biochar were observed through Scanning Electron Microscopy (SEM) analysis (Fig. 4a1, 2, 3). BM exhibited a multiple layered surface structure likely due to the aggregation of husk during the pyrolysis process (Duman et al. 2018). In contrast, BS and BES have a surface where the shell of the grape berries was still recognizable. Most internal components (i.e., albumen and embryo, with high content of volatile components) were removed during pyrolysis (Jimenez-Cordero et al. 2013). BP1 and BP2 presented typical woody texture, with the remainder of punctuations and conducting vessels forming honeycomb-like patterns (Uysal et al. 2024).

Energy-dispersive X-ray spectroscopy (EDS) analysis showed that pristine biochar has high content of mineral elements (Table S9), especially K, Ca, and P, known to enhance soil fertility (Ibn Ferjani et al. 2019; Anđelini et al. 2023). BM and BP1 had the highest K content at 29.26 and 26.58% (w/w), respectively. BS expressed the highest content in Ca with 26.44% (w/w) and in P with 10.32 (w/w). Moreover, biochar presents concretions with these minerals in their surface (Fig. S6). The presence of other elements was observed at different proportions depending upon the type of biochar such as Mg (4.04% and 4.33% w/w in BS and BP2), Si (2.27% w/w in BM), or Fe (2.77% w/w in BM). Finally, other elements in trace amounts (< 1% w/w) were found in these biochar (Al, S, Cl, Na…) as identified via ion chromatography (Table S1).

#### Cu-loaded biochar

**Fig. 5:**
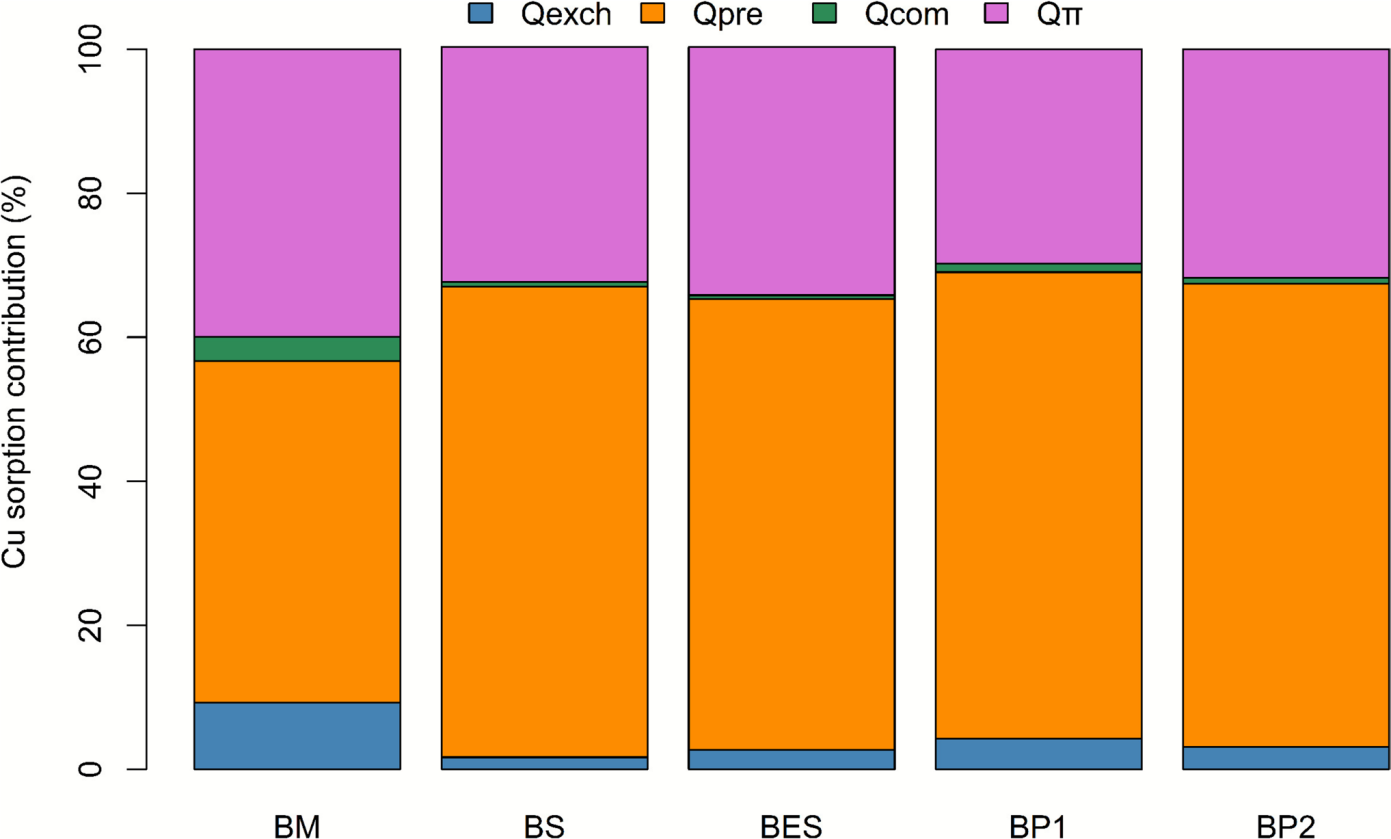
Quantitative contribution (%) of sorption mechanisms (*Q*_*exch*_: ion exchange, *Q*_*pre*_: precipitation, *Q*_*com*_: complexation, *Q*_*π*_: π-cation interactions) for each biochar type (BM, marc biochar; BS, seed biochar; BES, biochar extracted seed; BP1, pruning biochar; BP2, pruning biochar with two pyrolysis step)

EDS analysis confirmed the presence of Cu in Cu^2^⁺-loaded biochars (Fig. 4b8; Table S10), while no Cu was detected in pristine biochar (Fig. 4a4; Table S9), demonstrating effective Cu sorption. In comparison with pristine biochar, Cu-loaded biochar showed a decrease in K, Ca, and Mg contents, as also observed by Zhang et al. (2021). Interestingly, K content in BM decreased by 95%, suggesting strong ion exchange with K⁺ ions, consistent with the high relative contribution of ion exchange for BM (Fig. 5). Cu may co-precipitate with minerals as amorphous structure, copper phosphate, copper carbonate (Zhang et al. 2021), copper silicate, or hydrolyzed as Cu(OH)₂ (Meng et al. 2014). It can also be sorbed within pre-existing calcium structures or incorporated into new precipitates (Rees et al. 2017). SEM images showed Cu localized within mineral concretions on the biochar surfaces (Fig. S6), and the FT-IR analysis indicated bands possibly corresponding to PO₄^3^⁻ (~ 1040 cm⁻^1^) and CO₃^2^⁻ (~ 1415 cm⁻^1^) (Fig. 1), supporting the precipitation of copper phosphate or carbonate (Zhang et al. 2013). This is further supported by the high PO₄^3^⁻ content in biochar (up to 4812.2 ± 246.30 mg·kg⁻^1^ for BM; Table S1) and the significant contribution of precipitation in Cu sorption mechanisms across all biochars (Fig. 5).

### Copper sorption mechanisms

The relative contributions of the different Cu sorption chemical mechanisms across the five biochars are shown in Fig. 5 and associated statistical analysis in Table S1. These mechanisms included (1) π-electron interactions (*Q*_*π*_) with aromatic carbon structures, (2) precipitation (*Q*_*pre*_) with mineral phases such as carbonates or phosphates, considered stable, (3) complexation (*Q*_*com*_) with oxygen-containing functional groups (e.g., –COOH, –OH), and (4) cation exchange (*Q*_*exch*_) with exchangeable surface cations like K⁺, Ca^2^⁺, or Mg^2^⁺ which were considered less stable (Li et al. 2017; Qiu et al. 2022; Mei et al. 2025). Additionally, physical adsorption through pore filling exists; however, this is a reversible mechanism, not playing a decisive role in overall adsorption performance and, therefore, was not quantified (Mei et al. 2025). The relative contribution of each mechanism depended on biochar’s surface chemical composition and microstructure (Zhang et al. 2021); these information help in the prediction on Cu^2^⁺ desorption characteristics from biochars after adsorption (Liu et al. 2024).

Similar trends were observed for all biochars, with *Q*_*π*_ (29.78–39.94%) and *Q*_*pre*_ (65.34–47.42%) contributing most to total sorption, while *Q*_*com*_ remained the lowest (< 4%). *Q*_*exch*_, usually higher for divalent ions (Wu et al. 2021), stands third with 1.66–9.25%. The low amount of *Q*_*com*_ is explained by high-temperature pyrolysis (700 °C), which reduced the amount of O-rich functional groups (Tomczyk et al. 2020; Janu et al. 2021). Inversely, *Q*_*π*_ increased due to a greater amount of condensed aromatic structures (Hu et al. 2019), following high temperature pyrolysis. The high relative contribution of *Q*_*π*_ was also explained by H/C ratios < 0.3 for all biochars (Table 2), indicating high aromaticity. Additionally, Zhao et al. (2023) described that *Q*_*π*_ interaction was the main mechanism for Cu^2^⁺ sorption, whereas precipitation depended mainly on mineral content, varying with feedstock (Arán et al. 2016). BM exhibited the most balanced sorption profile, which likely underlies its superior copper sorption capacity, whereas other biochars were imbalanced, with > 60% sorption contribution from *Q*_*pre*_.

Regarding relative contributions, *Q*_*exch*_ was significantly highest for BM, likely due to its greater content of exchangeable cations (2–4 times more K⁺, Ca^2^⁺, Mg^2^⁺; Table S1) which played an essential role in the cation exchange process (Zhang et al. 2021). *Q*_*pre*_ participation was high for all biochars, consistent with their higher mineral content (PO₄^3^⁻, Ca^2^⁺, CO₃^2^⁻) and higher ash content (e.g., BM 27.49%; BP1 11.25%), which favors precipitation processes as previously reported (Wang et al. 2018). Indeed, XRD analysis before and after Cu sorption can show the formation of amorphous or carbonate precipitates in biochar surface (Bandara et al. 2020; Zhang et al. 2021). Nonetheless, FT-IR results (Fig. 1) indicate a possible formation of copper carbonates or phosphates. *Q*_*com*_ was more important for BP and especially for BM, whose feedstocks contained more cellulose and hemicellulose, providing COOH and OH functional groups during pyrolysis (Hassan et al. 2020), as reflected by high O content and O/C ratio (Table 2). Additionally, the higher BM pH (11.74; Table 2) compared to other feedstocks may have enhanced aqueous solution pH, leading to more electrostatic interaction (Fig. 3) and precipitation (Mei et al. 2025). *Q*_*π*_ accounted for a large share in seed-based biochars, consistent with their lignin-rich feedstocks (Table 1) producing more condensed aromatic C during pyrolysis (Demirba 2000; Sharma et al. 2004).

### Copper desorption from biochar

Four desorbing agents were tested to evaluate Cu stability on biochars (Table 3). H₂O and 0.1 M NaOH induced minimal desorption (< 5.75% and < 1.31%, respectively), whereas 0.1 M HCl and 0.1 M EDTA resulted in high desorption (> 44.57% and > 52.15%, respectively). Although similar desorption trends were observed across the desorbing agent, variation among the different biochars was significant, suggesting distinct Cu sorption mechanisms for each biochar type.

**Table 3:** Copper desorption rates (%) of biochar samples after 6-h contact with various desorbing agents (H₂O, 0.1 M HCl, 0.1 M NaOH, and 0.1 M EDTA). Lowercase letters indicate significant differences among biochars within the same desorbing agent; uppercase letters indicate significant differences among desorbing agents for the same biochar (*p* < 0.05)

Desorbing agent	BM		BS		BES		BP1		BP2	
H_2_O	0.05 ± 0.04	cD	1.09 ± 0.06	cC	1.50 ± 0.30	cC	2.97 ± 0.41	bB	5.75 ± 0.71	cA
HCl	44.34 ± 4.10	aB	29.52 ± 1.61	aC	35.37 ± 2.11	aC	55.97 ± 5.22	aA	44.57 ± 2.04	aB
NaOH	0.11 ± 0.01	bC	0.93 ± 0.16	cB	0.84 ± 0.15	cB	0.77 ± 0.22	cB	1.31 ± 0.20	dA
EDTA	38.52 ± 4.54	aB	22.26 ± 1.17	bD	25.88 ± 2.84	bCD	52.15 ± 8.66	aA	32.46 ± 3.35	bBC

Cu desorption with H₂O was negligible for BM (0.05%), especially when compared to 2.97% desorption percentage of BP1 or 5.75% for BP2, suggesting a contribution of pore filling, a weak and reversible sorption mechanisms in pruning’s biochars (Mei et al. 2025). NaOH desorption was again minimal for BM (0.11%), while other biochars ranged from 0.77% to 1.31%, indicating Cu stability under alkaline conditions. This aligns with the Cu precipitation potential with hydroxyl groups at high pH, thereby limiting desorption in water (Batool et al. 2017). In the case of EDTA, due to its strong chelating ability, desorption is promoted by the formation of stable complexes with heavy metals at the biochar surface (Liu et al. 2024). With 0.1 M EDTA, BS and BES showed significantly lower desorption rates than the other biochars, suggesting a desorption process less influenced by EDTA—likely due to a greater contribution of ion exchange or π–cation interactions and a lower involvement of O-rich functional groups in Cu sorption (Zhang et al. 2019; Zhu et al. 2020).

The high desorption observed with 0.1 M HCl (up to 55.97% for BP1) likely resulted from alterations of oxygen-containing functional groups and the dissolution of Cu-rich precipitates under acidic conditions (Zhang et al. 2019; Liu et al. 2024). These results suggested that these biochars can be regenerated and reused in aqueous environments (Kołodyńska et al. 2017). In contrast, the low desorption observed with water—particularly for BM—indicates strong Cu retention, making BM more suitable for long-term Cu immobilization in soil remediation (Meng et al. 2022). Under field conditions, Cu may be mobilized through particulate erosion and enter aquatic systems (Banas et al. 2010). However, this study shows that when Cu is sorbed onto biochar, erosion of biochar particles into water may lead to only limited metal desorption.

As BM exhibited both the highest sorption capacity and greatest resistance to desorption, its sorption kinetics (10 min–24 h) were further examined (Table S12). Overall, the desorption of Cu from BM using the different desorbing agents exhibited a similar pattern to that observed in Table 3: very low (< 1%) with H₂O and NaOH, and > 50% with HCl and EDTA. According to the kinetics, only minor differences were observed, indicating that the fraction of Cu that can be desorbed is released rapidly, with no substantial increase over time, as reported in Chen et al. (2024), for example.

### Feedstocks and corresponding biochars phytotoxicity assessment

Phytotoxicity assessment (Table 1) revealed high toxicity in all feedstocks, with GM and GS completely inhibiting *Lepidium sativum* seed germination while P and EGS allowed limited growth (~ 25% of the control vigor index) though still exhibiting phytotoxicity. The observed toxicity is likely linked to their low pH (< 4.8 for GM and GS), whereas P and EGS showed slightly higher pH values (5.28 ± 0.06 and 4.9 ± 0.05 for P and EGS, respectively). The EC values followed a similar trend, reflecting high mineral content in feedstocks (Table S1). Additionally, GM toxicity may be attributed to its high fluoride content (4156 ± 87.4 mg·kg⁻^1^, ten times higher than in other feedstocks) (Panda 2015), as well as higher levels of zinc and lead (20–30 mg·kg⁻^1^), copper (> 200 mg·kg⁻^1^), and arsenic (2.93 ± 1.56 mg·kg⁻^1^) (Table S1), known to inhibit seed development (Bożym and Rybak 2024). Such phytotoxic effects of GM, including delayed seed germination, have been previously reported (Paradelo et al. 2012). Similar to its feedstock, BM was highly toxic (normalized SVI 4.9 ± 3.63) whereas the other biochars have much lower toxicity, although none reached control levels. The high toxicity of BM was attributed to its extreme pH (11.74 ± 0.01), high EC (15,789.6 ± 243 µS·cm⁻^1^), and ash content (27.49 ± 0.71%), which were significantly higher than those of the other four biochars. These parameters were mainly driven by high concentrations of metals and dissolved ions (Table S1), particularly copper, which was more abundant in BM (90.23 mg·kg⁻^1^) than in the other biochars (Visioli et al. 2016). SVI details are provided in Table S2. Additionally, biochar phytotoxicity is known to be dose-dependent (Li et al. 2015), and may also be attributed to polycyclic aromatic hydrocarbons (PAHs) (Gascó et al. 2016; De La Rosa et al. 2019) or volatile organic compounds such as acetone and benzene (El-Bassi et al. 2022).

### Statistical analysis of biomass and biochar properties

#### Pearson correlation matrix

As shown in Fig. S8, Cu sorption capacity (*Q*_*e*_) was strongly positively correlated with pH, EC, CEC, ash content, and mineral nutrients, in line with Thomas et al. (2020) findings. Mineral richness was also positively associated with pH, EC, and ash content, but negatively correlated with phytotoxicity (SVI, germination, radicle length) in line with biochar phytotoxicity explanation. Carbon content (% C) showed a positive correlation with toxicity diminution and a negative correlation with both oxygen content and the O/C ratio as expected (Hassan et al. 2020). Furthermore, WHC was positively correlated with porosity, consistent with the data analysis (Table 2).

#### Principal component analysis (PCA) on biochar characteristics

The first two principal components explained 86.3% of the total variance. PC1 (64.5%) was positively associated with pH, electrical conductivity (EC), ash content, oxygen content (% O), O/C ratio, and nutrient concentrations (K⁺, Ca^2^⁺, PO₄^3^⁻, Mg^2^⁺), as well as sorption capacity (*Q*_*e*_). It was negatively correlated with biological performance indicators (e.g., germination metrics) and combustion-related traits such as carbon content (% C) and heating values. PC2 (21.8%) distinguished biochars based on their physical structure, separating highly porous materials (characterized by elevated WHC, porosity, *S*_*BET*_, and volatile matter) from less stable, denser biochars, which exhibited higher H:C ratios (indicating lower aromatization) and greater bulk density.

**Fig. 6:**
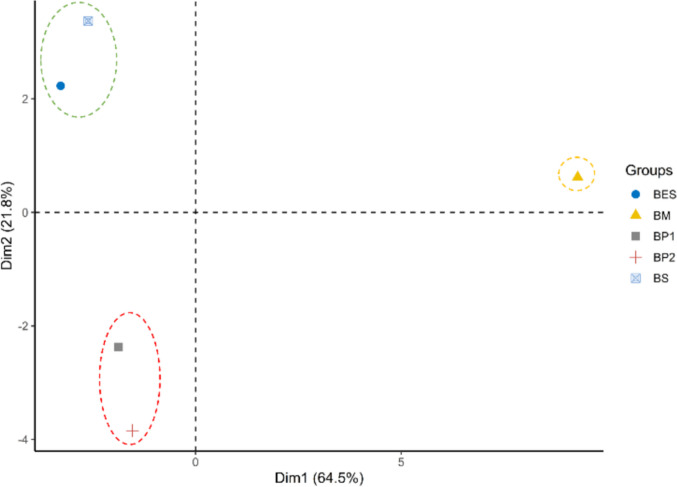
Principal component analysis (PCA) showing projection of biochars onto the first two principal components (Dim1 and Dim2). Ellipses represent clusters of similar biochars (BM, marc biochar; BS, seed biochar; BES, biochar extracted seed; BP1, pruning biochar; BP2, pruning biochar with two pyrolysis step)

The projection of modalities on the first two components (Fig. 6) and clustering revealed distinct groups of biochars. The BM cluster (yellow) was characterized by a high mineral ash content, leading to elevated pH and EC (strongly positive on PC1), but also exhibited marked phytotoxicity, placing it away from the biologically favorable region (negative on PC1). Such biochar likely has strong sorption potential via precipitation mechanisms and oxygen-rich surface functionalities, yet may pose toxicity risks at high application rates. The green cluster (top left), with BP1 and BP2 from similar feedstocks, showed low phytotoxicity due to reduced mineral content and was associated with enhanced structural properties, such as high WHC and porosity, making these biochars the most promising candidates in terms of agronomic performance. The red cluster (bottom left), including BS and BES derived from vines seeds, also showed low toxicity but had lower porosity, leading to intermediate agronomic potential. However, pruning-derived biochars (BP1/BP2) were located closer to the positive PC1 axis than seed-derived ones, suggesting a higher sorption capacity.

### Perspective of using biochar in circular bioeconomy

The feasibility of using biochar largely depends on the biomass’s availability, as well as the costs of transportation, storage, preprocessing, pyrolysis, and its final application (Da Silva et al. 2024). This study suggests an environmentally friendly strategy for managing vineyard by-products in the context of the copper contamination. As reported by the OIV, France ranks as the second-largest wine producer worldwide, with 36.1 million hectoliters in 2024 (https://www.oiv.int/fr), but also faces soil copper contamination derived from fungal control (Komárek et al. 2010). The Centre-Val de Loire region represents 8.5% of French vineyards and alone generates nearly 43,000 tons DW of grape marc—representing about 25% of the fresh biomass and composed of approximately 70% skins, 18% seeds, and 12% stalks (Guardia et al. 2018)—while vine pruning residues amount to 67,000 tons DW, providing a promising locally sourced biomass feedstock (IFV 2022). To reduce transportation costs, the use of mobile pyrolysis units has been proposed (Rosas et al. 2015). Converting biomass into biochar represents a “win–win” approach, improving waste management while protecting the environment (Tan et al. 2015). Indeed, the economic viability of biochar made from vineyard by-products, both alone and in co-production of syngas and other valuable compounds, has been demonstrated (Zabaniotou et al. 2018; Agraso-Otero et al. 2025), in line with new EU regulations (*EU 2019/1009*). This has also been shown by LCA analysis on vineyard by-products biochar (Ramos and Ferreira 2022; Simões et al. 2023). Beyond soil remediation, biochar therefore fits into a circular bioeconomy model, where the co-production of energy and materials enhances resource efficiency, supports climate change mitigation, and contributes to sustainable viticulture.

## Conclusion

This study aimed to evaluate the effectiveness of biochars derived from viticultural by-products for copper adsorption applications. The specific objectives were (1) to examine how feedstock type affects biochar physicochemical properties, (2) to investigate copper adsorption and desorption capacities of the resulting biochars, and (3) to identify the underlying mechanisms driving copper sorption.

The main findings were as follows:

1. Feedstock origin significantly influenced the physicochemical properties of the biochars, primarily due to differences in the initial composition of hemicelluloses, cellulose, and lignin.
2. These variations led to distinct sorption capacities, with the following performance: BM > BP1/BP2 > BS/BES. BM exhibited the highest Cu sorption capacity (*Q*_*max*_ 128.21 mg_Cu_·g⁻^1^), best described by Langmuir isotherm and PSO kinetics models.
3. Main copper sorption mechanisms were precipitation and π–cation interactions, as supported by complementary analyses including FT-IR spectroscopy, zeta potential measurements, SEM–EDS imaging, and ion analysis.
4. Desorption remained minimal under water leaching and 1.0 M NaOH extraction, indicating a strong retention of copper.

These results confirmed previous observations regarding the efficiency of biochar in trace metal sorption and its limited desorption potential (Da Silva et al. 2024; Y. Jin et al. 2021; H. Li et al. 2018; Trakal et al. 2014). Overall, biochars derived from viticultural residues demonstrated significant potential for use as sustainable remediation materials for copper-contaminated vineyard soils, thereby supporting circular economy strategies and contributing to soil remediation and climate change mitigation.

In addition to their sorption performance, the biochars also exhibited agronomic benefits, including improved water retention (notably with BP1 and BP2), a fertilizing effect (particularly with BM), and carbon sequestration in soil, all of which contributed to climate change mitigation.

Further studies may investigate the effect of Cu sorption by biochar in contaminated vineyard soil while assessing their impact on plants and microorganisms. Additionally, modifications of biochars to increase their Cu sorption capacity in solution, as well as the effect of pH and copper formulation on sorption performances should also be considered.

## Supplementary Information

Below is the link to the electronic supplementary material.

ESM 1 (DOCX 4.25 MB)

## Data Availability

Data are available upon reasonable request.
