# Supplementary material for: Comprehensive characterization of viticultural biomass and its derived biochars: insights into copper sorption potential
Source: Environ Sci Pollut Res Int. 2026 Mar 6;33(10):4405–28. doi: 10.1007/s11356-026-37550-0 (PMC13053484; doi:10.1007/s11356-026-37550-0)
Supplement: Supplementary file 1 — (DOCX 4.25 MB) [file 11356_2026_37550_MOESM1_ESM.docx]

**Comprehensive Characterization of Viticultural Biomass and Its Derived Biochars: Insights into Copper Sorption Potential**

Hugo Henaut^1^, Yassine Chafik^1^, Marta Sena-Velez^1^, Benoît Cagnon^2^ Sylvain Bourgerie^1+^ and Domenico Morabito^1+^

^1^Université d’Orléans, P2E, UR 1207 - USC INRAé 1328, Orléans, France.

^2^Université d’Orléans, CNRS, ICMN, UMR 7374, Orléans, France.

^+^ These authors contributed equally to this work

HH: [hugo.henaut@univ-orleans.fr](mailto:hugo.henaut@univ-orleans.fr); YC: [yassine.chafik@univ-orleans.fr](mailto:yassine.chafik@univ-orleans.fr); MSV: [marta.sena-velez@univ-orleans.fr](mailto:marta.sena-velez@univ-orleans.fr); BC: [benoit.cagnon@univ-orleans.fr](mailto:benoit.cagnon@univ-orleans.fr); SB: [sylvain.bourgerie@univ-orleans.fr](mailto:sylvain.bourgerie@univ-orleans.fr); DM: [domenico.morabito@univ-orleans.fr](mailto:domenico.morabito@univ-orleans.fr)

*Corresponding author email address and phone number:* [*domenico.morabito@univ-orleans.fr*](mailto:domenico.morabito@univ-orleans.fr)*; +33(0)2 38 41 72 35*

**Supplementary Information**

**Additional Tables and Figures**







**



**

**Fig S1** Thermogravimetric (TG, mass loss %) and Differential Thermogravimetric (DTG) analysis of feedstocks over 30–750°C: (A) Grape Marc (GM), (B) Pruning (P), (C) Grape Seed (GS), (D) Extracted Grape Seed (EGS). Red line: TG; Blue line: DTG











**Fig S2** Differential Scanning Calorimetry (DSC, red line) and Differential Thermogravimetric (DTG, blue line) curves of feedstocks over 30–750°C: (**A)** GM: Grape Marc; (**B**) P: Pruning; (**C**) GS: Grape Seed; (**D**) EGS: Extracted Grape Seed


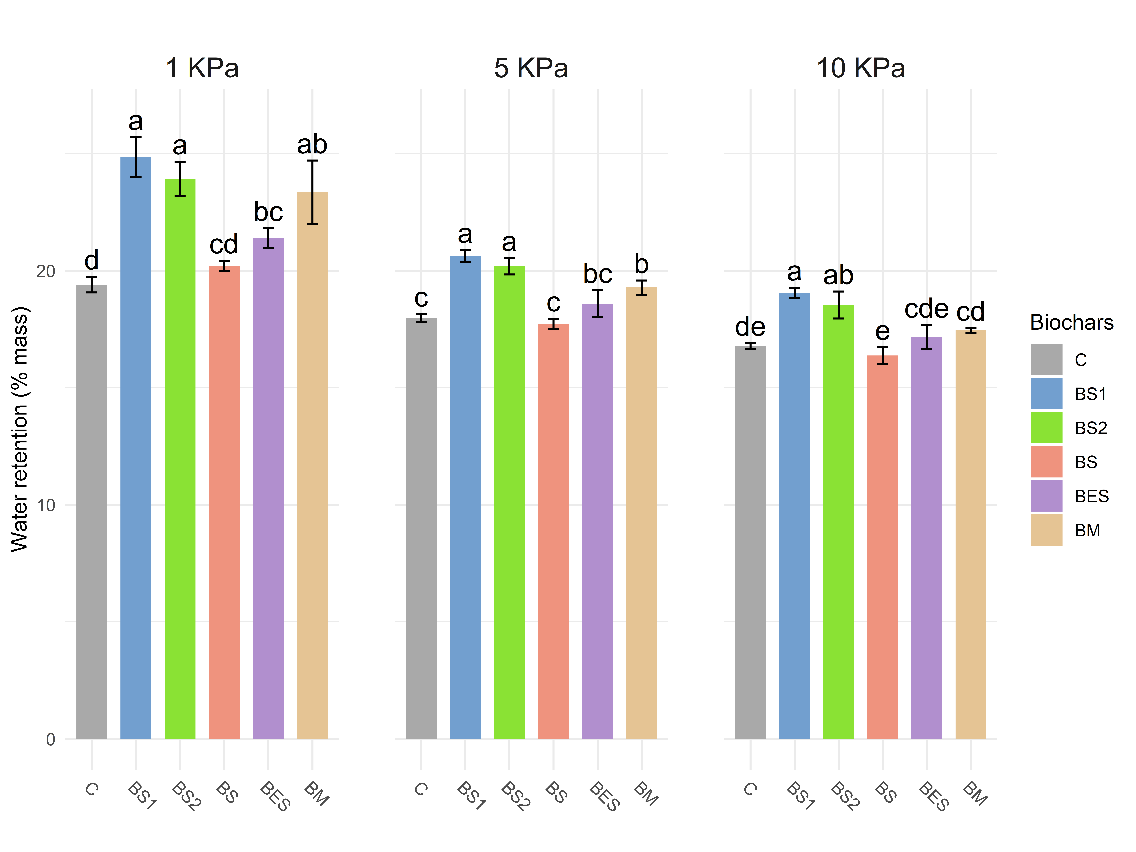


**Fig. S3** Water retention curves showing soil moisture (%) at 1–10 kPa suction for control soil and soils amended with 2% (w/w) of different biochars. Values are mean ± SD (*n* = 4). Letters indicate significant differences between treatments at each suction force (*p* < 0.05)





**Fig. S4** Van Krevelen diagram showing the atomic H/C and O/C ratios of feedstocks and their corresponding biochars, illustrating the progression of carbonization during pyrolysis

**Table S1** Major and minor elemental composition of feedstocks (GM, P, GS, EGS) and their corresponding biochars (BM, BS, BES, BP1, BP2). Results are in mg/kg; “<DL” indicates below detection limit. (GM: Grape Marc; P: Prunning; GS: Grape Seed; EGS: Extracted Grape Seed). and Biochar (BM: Marc Biochar; BS: Seed Biochar; BES: Biochar Extracted seed; BP1: Pruning Biochar; BP2: Pruning Biochar with two pyrolysis)

| Feedstock | GP | P | GS | EGS |  | Biochar | BM | BP1 | BP2 | BS | BES |
| --- | --- | --- | --- | --- | --- | --- | --- | --- | --- | --- | --- |
| Major Elements (mg.kg^-1^) |  |  |  |  |  | Major Elements (mg.kg^-1^) |  |  |  |  |  |
| F^-^ | 4156 ± 87.4 | 293.3 ± 6.1 | 968.5 ± 28 | 417.8 ± 6.4 |  | F^-^ | 115.2 ± 5.2 | <DL | <DL | 16.2 ± 2 | 11.7 ± 1.7 |
| Cl^-^ | 237.7 ± 8.3 | 154.4 ± 5.2 | 115.8 ± 2.2 | 175.9 ± 0.7 |  | Cl^-^ | 536.2 ± 8.3 | 466.2 ± 5.7 | 566.3 ± 13 | 232.6 ± 0.8 | 183.8 ± 1.2 |
| NO_2_^-^ | <DL | <DL | <DL | <DL |  | NO_2_^-^ | 62.9 ± 24.8 | <DL | <DL | <DL | <DL |
| Br^-^ | <DL | <DL | <DL | <DL |  | Br^-^ | <DL | <DL | <DL | <DL | <DL |
| NO_3_^-^ | 257.3 ± 28 | 91.1 ± 10.6 | 77.9 ± 2.1 | 242.1 ± 7.2 |  | NO_3_^-^ | 47.7 ± 21.2 | 43.9 ± 20.4 | 44.9 ± 20.4 | 38.9 ± 16.9 | 40.3 ± 18 |
| PO_4_^3-^ | 5572.1 ± 111.9 | 2293.4 ± 46.1 | 2611 ± 3.4 | 7976.1 ± 252.8 |  | PO_4_^3-^ | 4812.2 ± 246.3 | 163.6 ± 22.5 | 90.1 ± 19 | 456.3 ± 20.6 | 671.7 ± 19.6 |
| SO_4_^2-^ | 857.6 ± 70.8 | 235.2 ± 5.8 | 354.1 ± 10.2 | 190.7 ± 35.9 |  | SO_4_^2-^ | 888.6 ± 26.5 | 998.6 ± 14.7 | 222.8 ± 25.5 | 90.4 ± 26.7 | 78.5 ± 25.1 |
| Li^+^ | 0.58 ± 0.58 | 0.74 ± 0.27 | 1.14 ± 0.84 | 1.23 ± 0.7 |  | Li^+^ | <DL | <DL | <DL | 0.01 ± 0.02 | 0.01 ± 0.01 |
| Na^+^ | 111.01 ± 28.65 | 148.08 ± 5.8 | 57.5 ± 10.1 | 88.44 ± 9.73 |  | Na^+^ | 821.65 ± 7.92 | 122.45 ± 0.88 | 141.73 ± 0.4 | 161.54 ± 143.07 | 106.26 ± 14.59 |
| NH_4_^+^ | 647.21 ± 70.92 | 98.02 ± 49.86 | 196.83 ± 36.47 | 58.25 ± 24.8 |  | NH_4_^+^ | <DL | <DL | <DL | <DL | <DL |
| K^+^ | 20510.18 ± 2528.25 | 4500.42 ± 787.56 | 6612.87 ± 741.92 | 5997.69 ± 676.85 |  | K^+^ | 41824.8 ± 262.8 | 11760.36 ± 73.89 | 8895.95 ± 11.65 | 2738.35 ± 1804.87 | 2267.13 ± 309.19 |
| Mg^2+^ | 666.6 ± 69.75 | 295.49 ± 60.7 | 1001.51 ± 101.43 | 1190.82 ± 116.01 |  | Mg^2+^ | 19.35 ± 16.82 | 9.26 ± 0.41 | 9.08 ± 0.62 | 3.42 ± 4.22 | 5.21 ± 1.29 |
| Ca^2+^ | 894.99 ± 112.63 | 825.4 ± 83.08 | 745.98 ± 11.85 | 706.85 ± 29.53 |  | Ca^2+^ | 120.48 ± 1.04 | 31.02 ± 0.19 | 20.21 ± 0.2 | 15.95 ± 15.56 | 20.98 ± 2.7 |
| Minor Elements (mg.kg^-1^) |  |  |  |  |  | Minor Elements (mg.kg^-1^) |  |  |  |  |  |
| Cu | 205.11 ± 75.94 | 150.8 ± 19.44 | 109.09 ± 30.38 | 103.52 ± 12.76 |  | Cu | 90.23 ± 1.16 | 33.47 ± 3.06 | 31.02 ± 2.78 | 30.54 ± 0.72 | 19.27 ± 1.98 |
| Zn | 21.59 ± 5.47 | 16.13 ± 3.33 | 17.55 ± 3.55 | 15.39 ± 1.87 |  | Zn | 49.41 ± 4.44 | 66.12 ± 13.07 | 41.7 ± 2.57 | 24.78 ± 2.89 | 27.04 ± 4.22 |
| Pb | 29.42 ± 56.49 | <DL | <DL | <DL |  | Pb | 1.44 ± 1.08 | <DL | 0.52 ± 0.08 | 0.37 ± 0.38 | <DL |
| Mn | 28.91 ± 4.16 | 18.35 ± 1.21 | 17.26 ± 1.24 | 16.35 ± 0.85 |  | Mn | 123.58 ± 18.75 | 92.81 ± 15.34 | 79.68 ± 4 | 44.37 ± 6.19 | 52.26 ± 6.15 |
| Fe | 227.36 ± 40.79 | 57.66 ± 9.88 | 198.85 ± 24.72 | 116.07 ± 7.58 |  | Fe | 1098.66 ± 199.02 | 956.69 ± 167.53 | 1407.26 ± 262.33 | 484 ± 218.15 | 925.52 ± 539.44 |
| Cd | <DL | <DL | <DL | <DL |  | Cd | 0.22 ± 0.02 | 0.13 ± 0.02 | 0.13 ± 0.05 | 0.1 ± 0.01 | 0.11 ± 0.01 |
| As | 2.93 ± 1.56 | 2.33 ± 0.16 | 1.85 ± 0.73 | 1.86 ± 0.73 |  | As | 4.82 ± 0.47 | 2.41 ± 0.42 | 3.27 ± 0.19 | 2.18 ± 0.3 | 2.15 ± 0.21 |

**Table. S2** Seed germination parameters for *Lepidium sativum* exposed to aqueous extracts from feedstocks and biochars. Includes Germination Rate (GR), Radicle Length (RL), and Seed Vigor Index (SVI). Values are mean ± SD (*n* = 3); letters indicate significant differences (*p* < 0.05) (GM: Grape Marc; P: Prunning; GS: Grape Seed; EGS: Extracted Grape Seed) and biochars (BM: Marc Biochar; BS: Seed Biochar; BES: Biochar Extracted seed; BP1: Pruning Biochar; BP2: Pruning Biochar with two pyrolysis)

|  |  |  |  |  |  |  |
| --- | --- | --- | --- | --- | --- | --- |
| Feedstock | GR (%) |  | RL (cm) |  | SVI |  |
| GM | 0.0 ± 0.0 | c | 0.0 ± 0.0 | c | 0.0 ± 0.0 | c |
| P | 40 ± 14.1 | b | 8.6 ± 0.69 | b | 337.5 ± 90.32 | b |
| GS | 0.0 ± 0.0 | c | 0.0 ± 0.0 | c | 0.0 ± 0.0 | c |
| EGS | 50 ± 16.3 | b | 8.15 ± 1.58 | b | 397.5 ± 123.39 | b |
| Control | 92.5 ± 5 | a | 15.92 ± 2.86 | a | 1477.5 ± 309.34 | a |
| Biochar | GR (%) |  | RL (cm) |  | SVI |  |
| BM | 13.33 ± 11.55 | b | 2 ± 1.41 | c | 40 ± 34.64 | c |
| BP1 | 86.67 ± 15.28 | a | 9.53 ± 1.69 | b | 833.33 ± 256.97 | ab |
| BP2 | 76.67 ± 11.55 | a | 8.77 ± 1.66 | b | 680 ± 220.68 | b |
| BS | 76.67 ± 5.77 | a | 9.95 ± 1.8 | b | 760.74 ± 172.05 | b |
| BES | 80 ± 10 | a | 11.17 ± 1.65 | ab | 880 ± 60.83 | ab |
| Control | 83.33 ± 5.77 | a | 11.41 ± 0.52 | ab | 953.33 ± 119.3 | a |

**Table S3** Literature review summary of biochars produced from grape-derived feedstocks via pyrolysis (500–800°C). Data include elemental composition and physicochemical properties, presented as mean ± SD and range

| Feedstocks | Parameters | Mean ± SD | Range of value | *Authors* |
| --- | --- | --- | --- | --- |
| GRAPE MARC | %C | 73.42 ± 13.78 | 56.2 - 96.03 | (Encinar et al. 1996; Demiral and Ayan 2011; Manolikaki et al. 2016; Volpe et al. 2018; Zabaniotou et al. 2018; Duman et al. 2018; Ibn Ferjani et al. 2019; Vamvuka et al. 2020; Ismail et al. 2020; Jin et al. 2020; Ferreira et al. 2021; Frikha et al. 2021; Lin et al. 2021; Yoon et al. 2021; Petrova et al. 2023) |
|  | %H | 2.47 ± 0.7 | 1.19 - 3.52 |  |
|  | %N | 2.01 ± 0.55 | 1.4 - 3.1 |  |
|  | %O* | 18.23 ± 8.63 | 4.76 - 34.9 |  |
|  | HHV (Mj.kg-1)** | 24.09 ± 2.04 | 17.78 - 28.06 |  |
|  | LHV (Mj.kg-1)** | 23.56 ± 2.04 | 19.87 - 27.81 |  |
|  | H/C | 0.4 ± 0.12 | 0.16 - 0.61 |  |
|  | O/C | 0.19 ± 0.1 | 0.04 - 0.44 |  |
|  | pH | 10.05 ± 0.75 | 8.8 - 11.08 |  |
|  | EC (µs.cm^-1^) | 1475.83 ± 1524.07 | 528 - 3400 |  |
|  | CEC (cmol.kg^-1^) | 85.08 ± 105.72 | 6.15 - 205.2 |  |
|  | %WHC | 110 ± 0 | - |  |
|  | %FC | 65.51 ± 11.98 | 38.7 - 81.11 |  |
|  | %VM | 21.01 ± 6.47 | 11.1 - 36.5 |  |
|  | %Ash | 14.45 ± 11.75 | 3.89 - 48.39 |  |
|  | Sbet*** | 192.91 ± 73.04 | 43.5 - 293.27 |  |
| Feedstocks | Parameters | Mean ± SD | Range of value | *Authors* |
| GRAPE SEED | %C | 82.94 ± 5.57 | 64 - 90.3 | (Jimenez-Cordero et al. 2013; Ferreira et al. 2015; Fidalgo et al. 2015; Mena et al. 2017; De Almeida et al. 2023; Pardo et al. 2023) |
|  | %H | 1.98 ± 0.73 | 0.9 - 3.1 |  |
|  | %N | 1.82 ± 0.42 | 0.4 - 2.3 |  |
|  | %O* | 11.26 ± 3.58 | 4.7 - 21 |  |
|  | HHV (Mj.kg-1)** | 26.39 ± 1.42 | 21.55 - 27.81 |  |
|  | LHV (Mj.kg-1)** | 25.97 ± 1.48 | 20.89 - 27.62 |  |
|  | H/C | 0.29 ± 0.12 | 0.12 - 0.58 |  |
|  | O/C | 0.1 ± 0.04 | 0.04 - 0.25 |  |
|  | pH | - | - |  |
|  | EC (µs.cm^-1^) | - | - |  |
|  | CEC (cmol.kg^-1^) | - | - |  |
|  | %WHC | - | - |  |
|  | %FC | 64.56 ± 11.82 | 56.2 - 72.92 |  |
|  | %VM | 32.29 ± 7.37 | 37.5 - 27.08 |  |
|  | %Ash | 9.87 ± 1.56 | 8.6 - 12.42 |  |
|  | Sbet*** | 58.82 ± 41.62 | 13 - 124 |  |
| Feedstocks | Parameters | Mean ± SD | Range of value | *Authors* |
| GRAPE PRUNING | %C | 76.15 ± 5.24 | 67.69 - 90.4 | (Gómez et al. 2016; Tag et al. 2016; Marshall et al. 2017; Hamzenejad Taghlidabad and Sepehr 2018; Sfakiotakis and Vamvuka 2018; Gámiz et al. 2019; Marshall et al. 2019; Libutti et al. 2021; Gezović et al. 2022; Rivelli and Libutti 2022; Balmuk et al. 2023; Cárdenas-Aguiar et al. 2023; Khaledi et al. 2023;; Uysal et al. 2024) |
|  | %H | 2.43 ± 0.78 | 1.44 - 3.97 |  |
|  | %N | 1.17 ± 0.5 | 0.21 - 2.41 |  |
|  | %O* | 15.65 ± 6.09 | 3.09 - 25.23 |  |
|  | HHV (Mj.kg-1)** | 24.72 ± 1.71 | 21.88 - 28.6 |  |
|  | LHV (Mj.kg-1)** | 24.21 ± 1.65 | 21.44 - 28.17 |  |
|  | H/C | 0.38 ± 0.12 | 0.22 - 0.57 |  |
|  | O/C | 0.16 ± 0.07 | 0.03 - 0.27 |  |
|  | pH | 9.78 ± 0.75 | 8.5 - 11.31 |  |
|  | EC (µs.cm^-1^) | 4788.44 ± 6389.02 | 121.23 - 18200 |  |
|  | CEC (cmol.kg^-1^) | 29.6 ± 7.94 | 20.1 - 40.38 |  |
|  | %WHC | 177 ± 67.88 | 129 - 225 |  |
|  | %FC | 75.81 ± 9.08 | 64.3 - 90 |  |
|  | %VM | 17.81 ± 5.51 | 9.3 - 28.91 |  |
|  | %Ash | 9.16 ± 3.12 | 4.71 - 16.9 |  |
|  | Sbet*** | 291.9 ± 207.47 | 13.9 - 577 |  |
| * If missing, estimated by difference (100-%C-%H-%N) | | |  |  |
| ** HHV and LHV were recalculated according to Frikha et al. (2021)*.* | | |  |  |
| ** too low values excluded (<10m²/g) | |  |  |  |

**Table S4** Langmuir and Freundlich isotherm parameters for Cu²⁺ sorption on biochars. The reported parameters include the maximum sorption capacity (*Q_max_* , mg·g⁻¹), the Langmuir constant (*K_L_*, L·mg⁻¹), the Freundlich constants (*K_F_*, (mg·g⁻¹)(L·mg⁻¹)ⁿ; *n*), and the regression coefficients (R²)

| Biochar | Model | Parameter | Value | R² |
| --- | --- | --- | --- | --- |
| BM | Langmuir | Q_max_ | 134.36 | 0.88 |
|  |  | K_L_ | 0.06 |  |
|  | Freundlich | n | 3.84 | 0.82 |
|  |  | K_F_ | 25.81 |  |
| BS | Langmuir | Q_max_ | 44.03 | 0.93 |
|  |  | K_L_ | 0.003 |  |
|  | Freundlich | n | 2.29 | 0.92 |
|  |  | K_F_ | 1.46 |  |
| BES | Langmuir | Q_max_ | 47.84 | 0.95 |
|  |  | K_L_ | 0.003 |  |
|  | Freundlich | n | 2.09 | 0.93 |
|  |  | K_F_ | 1.17 |  |
| BP1 | Langmuir | Q_max_ | 56.41 | 0.89 |
|  |  | K_L_ | 0.006 |  |
|  | Freundlich | n | 3.97 | 0.93 |
|  |  | K_F_ | 7.91 |  |
| BP2 | Langmuir | Q_max_ | 45.77 | 0.91 |
|  |  | K_L_ | 0.007 |  |
|  | Freundlich | n | 3.32 | 0.92 |
|  |  | K_F_ | 4.71 |  |







**Fig S5** (**A**) Cu²⁺ sorption capacities of marc biochar (BM) fitted with isotherm models: Langmuir and Freundlich; and (**B**) adsorption kinetics of Cu²⁺ onto BM biochar fitted with kinetic models: pseudo-first-order (PFO), pseudo-second-order (PSO), Elovich, and intraparticle diffusion models (*n* = 4 ± SD)

**Table S5** Langmuir and Freundlich isotherm parameters for Cu²⁺ sorption on marc biochar (BM). The reported parameters include the maximum sorption capacity (*Q_max_* , mg·g⁻¹), the Langmuir constant (*K_L_*, L·mg⁻¹), the Freundlich constants (*K_F_*, (mg·g⁻¹)(L·mg⁻¹)ⁿ; *n*), and the regression coefficients (R²)

| Model | Parameter | Value | R² |
| --- | --- | --- | --- |
| Langmuir | Q_max_ | 128.21 | 0.83 |
|  | K_L_ | 0.07 |  |
| Freundlich | n | 3.52 |  |
|  | K_F_ | 22.77 | 0.78 |

**Table S6** Evolution of solution pH, electrical conductivity (EC), and Cu²⁺ adsorption capacity (*Q_e_*) during sorption tests with marc biochar (BM). Values are mean ± SD (*n* = 5); letters indicate significant differences (*p* < 0.05)

| Cu (g.L^-1^) | pH |  | EC |  | Qe (mgCu.g^-1^) |  |
| --- | --- | --- | --- | --- | --- | --- |
| 0.05 | 10.72 ± 0.01 | a | 3148.14 ± 35.28 | h | 5.58 ± 0.09 | j |
| 0.10 | 10.58 ± 0.03 | b | 3268 ± 264.75 | gh | 10.45 ± 0.34 | ij |
| 0.20 | 10.42 ± 0.01 | c | 3531.86 ± 162.06 | fg | 20.58 ± 0.6 | hi |
| 0.25 | 10.23 ± 0.03 | d | 3411.82 ± 191.55 | gh | 26.03 ± 0.4 | gh |
| 0.30 | 9.95 ± 0.12 | e | 3070.64 ± 365.16 | h | 33.37 ± 1.24 | fg |
| 0.40 | 9.84 ± 0.18 | e | 3522.12 ± 71.14 | fg | 41.62 ± 0.44 | ef |
| 0.50 | 9.06 ± 0.07 | f | 3853.02 ± 47.65 | ef | 52.31 ± 1.37 | de |
| 0.75 | 7.95 ± 0.14 | g | 4281.46 ± 130.99 | de | 75.89 ± 2.05 | d |
| 1.00 | 6.76 ± 0.16 | h | 4605.6 ± 141.14 | cde | 101.66 ± 4.13 | c |
| 1.25 | 6.43 ± 0.02 | i | 5225.22 ± 61.82 | bcd | 102.72 ± 7.36 | c |
| 1.50 | 6.26 ± 0.02 | j | 5558.94 ± 165.07 | abc | 115.99 ± 2.56 | b |
| 2.00 | 6 ± 0.05 | k | 6940.52 ± 102.67 | ab | 123.06 ± 11.11 | ab |
| 2.50 | 5.68 ± 0.04 | l | 8380.5 ± 47.26 | a | 130.73 ± 6.1 | a |

**Table S7** Kinetic parameters and R² values of Cu²⁺ adsorption onto marc biochar (BM) at room temperature, fitted using first and second pseudo-order (PFO, PSO), Elovich, and intraparticle diffusion models. The reported parameters include the equilibrium adsorption capacity (*Q_e_*, mg·g⁻¹), the kinetic constants of the pseudo-first-order (k₁, min⁻¹) and pseudo-second-order (k₂, g·mg⁻¹·min⁻¹) models, the Elovich constants (α, mg·g⁻¹·min⁻¹; β, g·mg⁻¹), the intraparticle diffusion parameters (*K_diff_*, mg·g⁻¹·min⁻¹/²; C, mg·g⁻¹), and the regression coefficients (R²)

| Model | Parameter | Value | R² |
| --- | --- | --- | --- |
| PFO (Pseudo First Order) | K_1_ | 0.0014 | 0.33 |
|  | Q_e_ | 25.76 |  |
| PSO (Pseudo Second Order) | K_2_ | 114.94 | 0.85 |
|  | Q_e_ | 0.0007 |  |
| Elovich | β | 0.0803 | 0.69 |
|  | α | 267.03 |  |
| Intraparticle Diffusion | K_diff_ | 1.56 | 0.4 |
|  | C | 74.51 |  |

**Table S8**. Temporal evolution of solution pH, electrical conductivity (EC), and Cu²⁺ adsorption capacity (*Q_e_*) during sorption kinetics tests. Values are mean ± SD (*n* = 5); letters indicate significant differences (*p* < 0.05)

| Time (min.) | pH |  | EC (µS.m^-1^) |  | Qe (mg.g^-1^) |  |
| --- | --- | --- | --- | --- | --- | --- |
| 5 | 5.2 ± 0.08 | e | 6933.8 ± 171.25 | a | 38.08 ± 9.96 | e |
| 10 | 5.24 ± 0.05 | de | 6989.26 ± 126.99 | a | 44.08 ± 11.92 | e |
| 15 | 5.32 ± 0.03 | cd | 7021.2 ± 52.8 | a | 89.75 ± 1.93 | d |
| 30 | 5.26 ± 0.04 | de | 6975.04 ± 48.96 | a | 92.46 ± 3.53 | cd |
| 45 | 5.28 ± 0.04 | cde | 6954.94 ± 286.51 | a | 93.99 ± 4.13 | bcd |
| 60 | 5.26 ± 0.03 | de | 6975.42 ± 97.75 | a | 98.04 ± 5.04 | bcd |
| 90 | 5.36 ± 0.04 | c | 7116.4 ± 187.09 | a | 104.03 ± 5.99 | abc |
| 120 | 5.92 ± 0.06 | a | 7051.44 ± 191.23 | a | 105.43 ± 4.26 | abc |
| 180 | 5.85 ± 0.02 | ab | 7026.04 ± 67.08 | a | 106.87 ± 5.21 | abc |
| 240 | 5.84 ± 0.03 | ab | 6994.18 ± 29.81 | a | 107.87 ± 2.56 | ab |
| 300 | 5.79 ± 0.02 | b | 6993.4 ± 100.96 | a | 107.53 ± 5.65 | ab |
| 360 | 5.82 ± 0.02 | ab | 6968.8 ± 93.31 | a | 110.21 ± 3.84 | ab |
| 720 | 5.79 ± 0.04 | b | 6969.92 ± 49.09 | a | 114.12 ± 4.26 | a |
| 1440 | 5.8 ± 0.05 | b | 6933.9 ± 80.06 | a | 116.26 ± 3.77 | a |


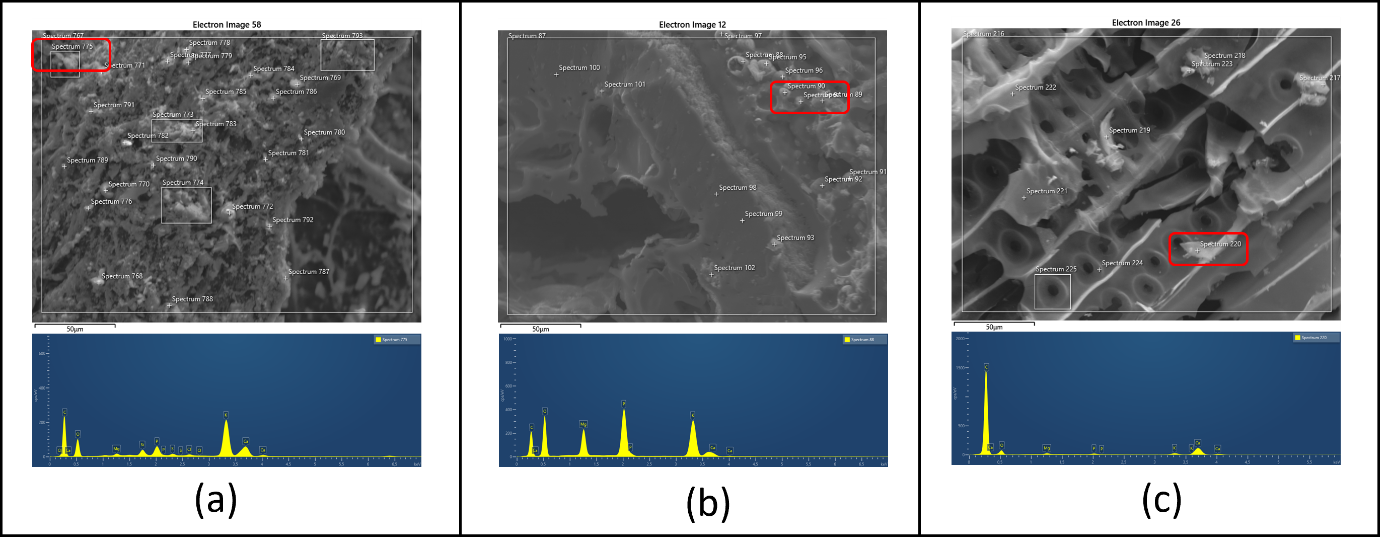


**Fig. S6:** SEM images and EDS spectra of selected red area showing mineral concretions in pristine biochars:
(a) BM (22.42% K, spectrum 775), (b) BS (12.01% P, spectrum 88), (c) BP1 (23.52% Ca, spectrum 220)


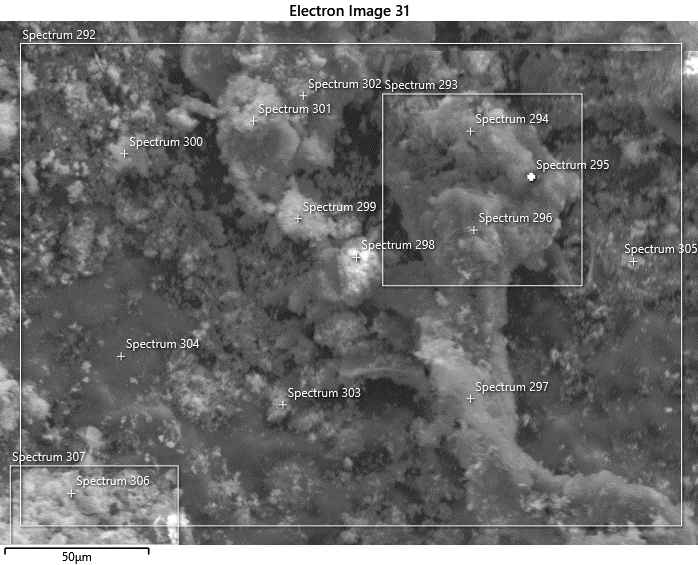

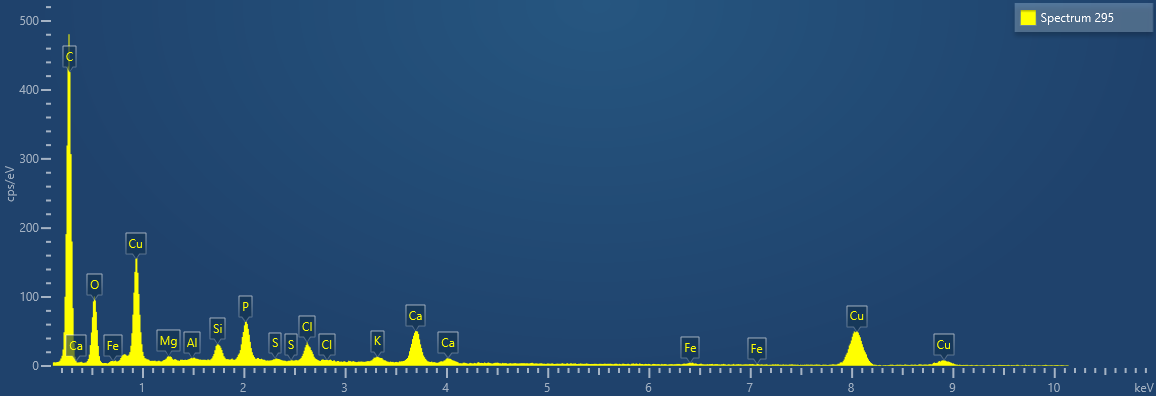

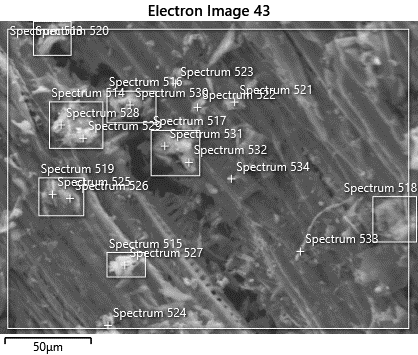

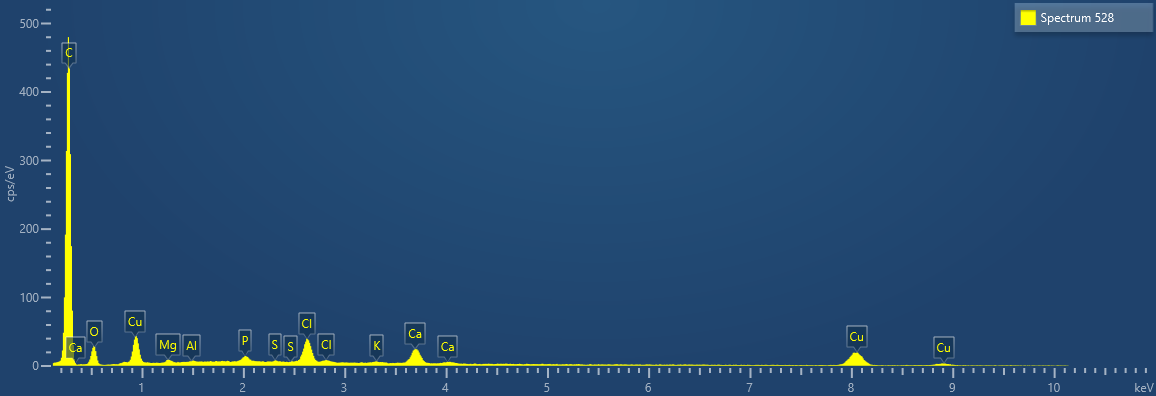

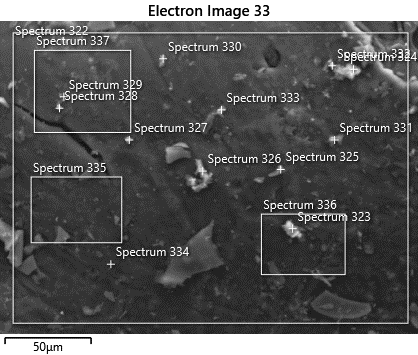

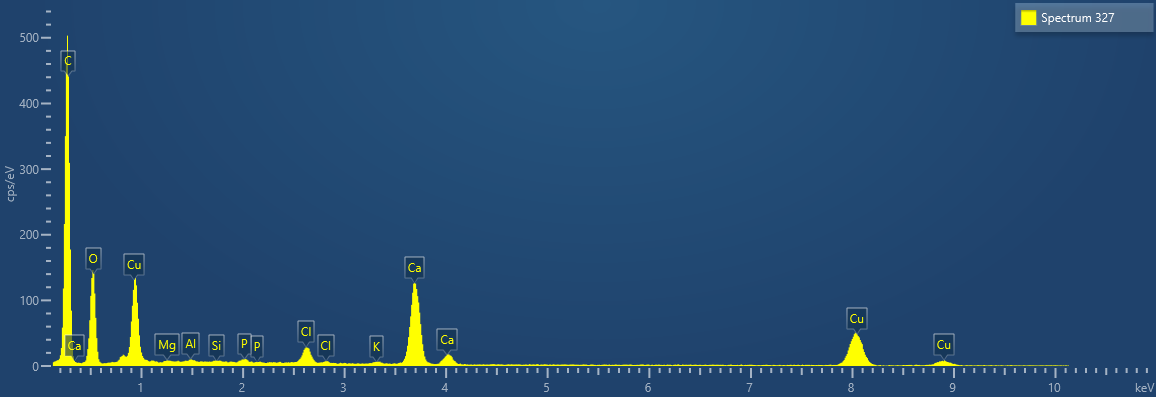


(a)

(c)

(b)

**Fig. S7:** SEM images and EDS spectra of selected red area showing Cu distribution on biochar surfaces after sorption: (a) BM (35.83% Cu, spectrum 295; associated with Ca & P), (b) BS (28.28% Cu, spectrum 327; associated with Ca), (c) BP1 (15.45% Cu, spectrum 528; associated with Ca & Mg)

**Table S9** : EDS elemental analysis (weight % and atomic %) of pristine biochars (BM: Marc Biochar; BS: Seed Biochar; BES: Biochar Extracted seed; BP1: Pruning Biochar; BP2: Pruning Biochar with two pyrolysis step)

| Element | BM | | BS | | BES | | BP1 | | BP2 | |
| --- | --- | --- | --- | --- | --- | --- | --- | --- | --- | --- |
|  | % weight | % atomic | % weight | % atomic | % weight | % atomic | % weight | % atomic | % weight | % atomic |
| O | 40.19 | 50.62 | 43.84 | 52.30 | 48.47 | 55.50 | 43.40 | 52.57 | 51.22 | 42.14 |
| C | 15.12 | 15.08 | 16.46 | 26.16 | 18.20 | 27.76 | 16.29 | 13.17 | 25.83 | 18.22 |
| Ca | 4.42 | 2.22 | 16.44 | 7.83 | 8.67 | 3.96 | 7.30 | 3.53 | 5.45 | 11.23 |
| P | 2.97 | 1.93 | 10.32 | 5.04 | 3.22 | 1.91 | 0.62 | 0.39 | 1.21 | 1.93 |
| K | 29.26 | 25.37 | 8.18 | 5.04 | 16.16 | 7.57 | 26.58 | 26.29 | 9.06 | 15.96 |
| Mg | 0.78 | 0.64 | 4.04 | 3.17 | 1.62 | 1.22 | 2.30 | 1.83 | 3.33 | 4.16 |
| Si | 2.27 | 1.00 | 0.39 | 0.26 | 1.02 | 0.67 | 0.72 | 0.50 | 0.35 | 0.50 |
| S | 1.03 | 0.64 | 0.23 | 0.14 | 0.79 | 0.26 | 1.23 | 0.74 | 2.12 | 3.49 |
| Cl | 0.40 | 0.27 | 0.11 | 0.06 | 0.09 | 0.05 | 0.01 | 0.01 | 0.90 | 1.64 |
| Al | 0.49 | 0.37 | - | - | 0.97 | 0.66 | 0.74 | 0.53 | 0.54 | 0.75 |
| Fe | 2.77 | 1.63 | - | - | 0.79 | 0.45 | 0.49 | 0.27 | - | - |
| Na | 0.31 | 0.23 | - | - | - | - | 0.33 | 0.17 | - | - |
| Cu | - | - | - | - | - | - | - | - | - | - |

**Table S10** : EDS elemental analysis (weight % and atomic %) of Cu-loaded biochars after sorption tests (BM: Marc Biochar; BS: Seed Biochar; BES: Biochar Extracted seed; BP1: Pruning Biochar; BP2: Pruning Biochar with two pyrolysis step).

| Element | BM | | BS | | BES | | BP1 | | BP2 | |
| --- | --- | --- | --- | --- | --- | --- | --- | --- | --- | --- |
|  | % weight | % atomic | % weight | % atomic | % weight | % atomic | % weight | % atomic | % weight | % atomic |
| O | 36.17 | 50.80 | 28.27 | 23.11 | 32.04 | 45.27 | 40.73 | 46.66 | 44.55 | 54.27 |
| C | 32.28 | 25.98 | 11.40 | 11.04 | 21.11 | 24.04 | 29.63 | 24.70 | 17.19 | 27.89 |
| Ca | 3.48 | 2.13 | 4.31 | 3.02 | 4.17 | 3.71 | 4.23 | 2.60 | 14.15 | 6.88 |
| P | 3.80 | 2.53 | 1.56 | 1.22 | 7.22 | 5.27 | 2.46 | 1.95 | 0.48 | 0.30 |
| K | 1.47 | 1.12 | 6.43 | 4.00 | 3.79 | 2.19 | 1.31 | 0.83 | 5.18 | 2.58 |
| Mg | 1.83 | 1.36 | 2.91 | 2.62 | 6.57 | 3.88 | 1.69 | 1.75 | 1.72 | 1.38 |
| Si | 1.94 | 1.46 | 3.49 | 2.91 | 1.68 | 1.35 | 0.25 | 0.22 | 0.23 | 0.16 |
| S | 0.66 | 0.46 | 1.20 | 0.91 | - | - | - | - | - | - |
| Cl | 3.68 | 2.33 | 9.29 | 6.38 | 8.82 | 5.63 | 7.91 | 5.48 | 5.52 | 3.03 |
| Al | 0.22 | 0.18 | 1.60 | 1.45 | 0.95 | 0.80 | - | - | 0.29 | 0.21 |
| Fe | 0.59 | 0.24 | 0.75 | 0.33 | 0.88 | 0.35 | - | - | 0.18 | 0.06 |
| Na | - | - |  |  | - | - | - | - | - | - |
| Cu | 13.89 | 11.41 | 28.81 | 43.02 | 12.77 | 7.51 | 11.79 | 15.81 | 10.51 | 3.23 |

**Table S11** Values and statistical differences among biochars and mechanisms. Lowercase letters indicate significant differences among biochars within the same mechanisms; uppercase letters indicate significant differences among mechanisms for the same biochar (*p* < 0.05). (*Q_exch_*: ion exchange, *Q_pre_*: precipitation, *Q_com_*: complexation, *Q_π_*: π-cation interactions) for each biochar type (BM: Marc Biochar; BS: Seed Biochar; BES: Biochar Extracted seed; BP1: Pruning Biochar; BP2: Pruning Biochar with two pyrolysis step)

| Biochar | Qexch (%) |  | Qpre (%) |  | Qcom (%) |  | Qπ (%) |  |
| --- | --- | --- | --- | --- | --- | --- | --- | --- |
| BM | 9.25 ± 0.95 | aC | 47.42 ± 0.16 | cA | 3.39 ± 0.66 | aD | 39.94 ± 0.72 | aB |
| BP | 1.66 ± 0.61 | cC | 65.34 ± 1.07 | aA | 0.31 ± 0.04 | cC | 32.69 ± 1.04 | bcB |
| BPE | 2.69 ± 0.72 | bcC | 62.60 ± 0.96 | bA | 0.24 ± 0.03 | cD | 34.48 ± 0.95 | bB |
| BS1 | 4.25 ± 1.00 | bC | 64.77 ± 1.09 | aA | 1.20 ± 0.33 | bD | 29.78 ± 0.89 | dB |
| BS2 | 3.13 ± 1.00 | bcC | 64.31 ± 1.07 | abA | 0.79 ± 0.15 | bD | 31.77 ± 1.16 | cdB |

**Table S12** Copper desorption rates (%) of marc biochar (BM) samples after 6-hour contact with various desorbing agents (H₂O, 0.1 M HCl, 0.1 M NaOH, and 0.1 M EDTA). Lowercase letters indicate significant differences among contact time within the same desorbing agent; uppercase letters indicate significant differences among desorbing agents for the same contact time (*p* < 0.05, *n* = 4)

| Contact time (min) | H2O |  | HCl |  | NaOH |  | EDTA |  |
| --- | --- | --- | --- | --- | --- | --- | --- | --- |
| 10 | 0.35 ± 0.08 | cdB | 73.19 ± 7.03 | Aa | 0.34 ± 0.02 | bcB | 54.85 ± 1.48 | abcA |
| 30 | 0.25 ± 0.01 | dB | 66.09 ± 3.49 | aA | 0.2 ± 0.07 | cB | 53.07 ± 1.93 | bcA |
| 60 | 0.33 ± 0.09 | cdB | 61.34 ± 2.92 | aA | 0.05 ± 0.02 | dB | 61.65 ± 3.66 | abA |
| 120 | 0.25 ± 0.03 | dC | 66.57 ± 2.98 | aA | 0.44 ± 0.05 | abB | 60.85 ± 3.61 | abcC |
| 240 | 0.47 ± 0.07 | bcC | 66.01 ± 6 | aA | 0.05 ± 0.01 | dD | 52.28 ± 2.35 | cB |
| 260 | 0.52 ± 0.02 | bC | 66.24 ± 5.88 | aA | 0.36 ± 0.15 | bcC | 53.99 ± 4.3 | abcB |
| 720 | 0.84 ± 0.12 | aB | 68.95 ± 9.34 | aA | 0.75 ± 0.07 | aB | 60.26 ± 4.8 | abcA |
| 1440 | 0,48 ± 0,06 | bcB | 73,01 ± 8,67 | aA | 0,69 ± 0,06 | aC | 62,66 ± 6,66 | aA |


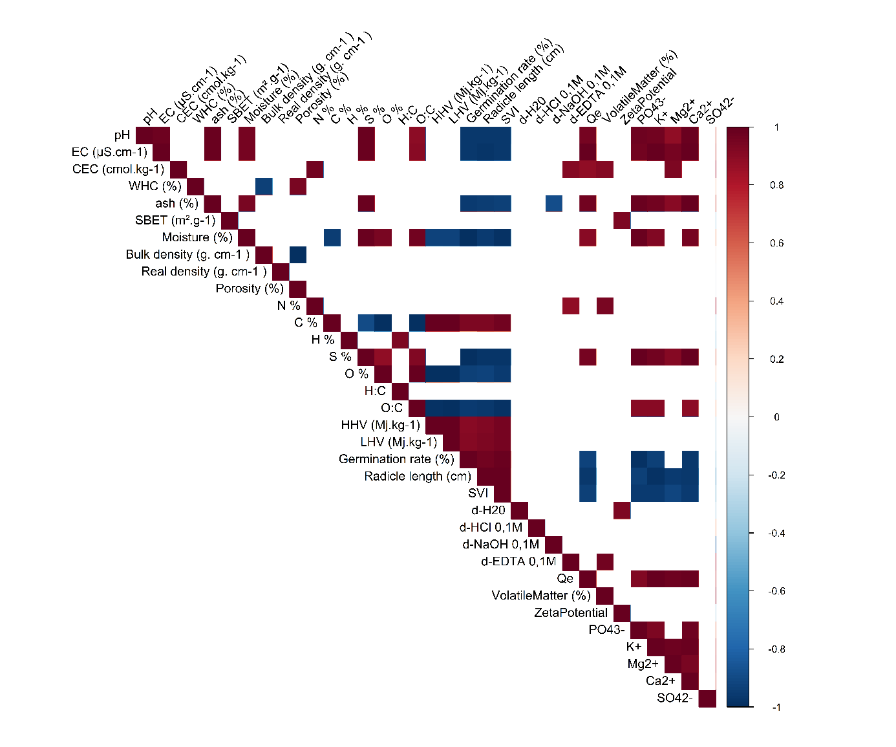


**Fig. S8.** Correlation matrix of biochar characteristics, showing significant correlations (*p* < 0.05). Positive correlations are shown in red, negative in blue; color intensity indicates correlation strength. (BM: Marc Biochar; BS: Seed Biochar; BES: Biochar Extracted seed; BP1: Pruning Biochar; BP2: Pruning Biochar with two pyrolysis step)

**References**

Balmuk G, Videgain M, Manyà JJ, Duman G, Yanik J (2023) Effects of pyrolysis temperature and pressure on agronomic properties of biochar. J Anal Appl Pyrolysis 169:105858. https://doi.org/10.1016/j.jaap.2023.105858

Cárdenas-Aguiar E, Gascó G, Lado M, Méndez A, Paz-Ferreiro J, Paz-González A (2023) New insights into the production, characterization and potential uses of vineyard pruning waste biochars. Waste Manag 171:452–462. https://doi.org/10.1016/j.wasman.2023.09.032

De Almeida LC, Andrade ELO, Santos JCB, Santos RM, Fricks AT, Freitas LDS, Lima ÁS, Pereira MM, Soares CMF (2023) Novel Nanobiocatalyst Constituted by Lipase from Burkholderia cepacia Immobilized on Graphene Oxide Derived from Grape Seed Biochar. C 9(1):12. https://doi.org/10.3390/c9010012

Demiral İ, Ayan EA (2011) Pyrolysis of grape bagasse: Effect of pyrolysis conditions on the product yields and characterization of the liquid product. Bioresour Technol 102(4):3946–3951. https://doi.org/10.1016/j.biortech.2010.11.077

Duman G, Tag AT, Ucar S, Yanik J (2018) Comparative evaluation of dry and wet carbonization of agro industrial wastes for the production of soil improver. J Environ Chem Eng 6(2):3366–3375. https://doi.org/10.1016/j.jece.2018.05.009

Encinar JM, Beltran FJ, Bernalte A (1996) Pyrolysis of two agricultural residues: olive and grape bagasse. Influence of particle size and temperature. Biomass Bioenergy 11(5):397–409

Ferreira AF, Ribau JP, Costa M (2021) A decision support method for biochars characterization from carbonization of grape pomace. Biomass Bioenergy 145:105946. https://doi.org/10.1016/j.biombioe.2020.105946

Ferreira CIA, Calisto V, Santos SM, Cuerda-Correa EM, Otero M, Nadais H, Esteves VI (2015) Application of pyrolysed agricultural biowastes as adsorbents for fish anaesthetic (MS-222) removal from water. J Anal Appl Pyrolysis 112:313–324. https://doi.org/10.1016/j.jaap.2015.01.006

Fidalgo B, Berrueco C, Millan M (2015) Chars from agricultural wastes as greener fuels for electric arc furnaces. J Anal Appl Pyrolysis 113:274–280. https://doi.org/10.1016/j.jaap.2015.01.027

Frikha K, Limousy L, Arif MB, Thevenin N, Ruidavets L, Zbair M, Bennici S (2021) Exhausted Grape Marc Derived Biochars: Effect of Pyrolysis Temperature on the Yield and Quality of Biochar for Soil Amendment. Sustainability 13(20):11187. https://doi.org/10.3390/su132011187

Gámiz B, Hall K, Spokas KA, Cox L (2019) Understanding Activation Effects on Low-Temperature Biochar for Optimization of Herbicide Sorption. Agronomy 9(10):588. https://doi.org/10.3390/agronomy9100588

Gezović A, Mišurović J, Milovanović B, Etinski M, Krstić J, Grudić V, Dominko R, Mentus S, Vujković MJ (2022) High Al-ion storage of vine shoots-derived activated carbon: New concept for affordable and sustainable supercapacitors. J Power Sources 538:231561. https://doi.org/10.1016/j.jpowsour.2022.231561

Gómez N, Rosas JG, Singh S, Ross AB, Sánchez ME, Cara J (2016) Development of a gained stability index for describing biochar stability: Relation of high recalcitrance index (R50) with accelerated ageing tests. J Anal Appl Pyrolysis 120:37–44. https://doi.org/10.1016/j.jaap.2016.04.007

Hamzenejad Taghlidabad R, Sepehr E (2018) Heavy metals immobilization in contaminated soil by grape-pruning-residue biochar. Arch Agron Soil Sci 64(8):1041–1052. https://doi.org/10.1080/03650340.2017.1407872

Ibn Ferjani A, Jeguirim M, Jellali S, Limousy L, Courson C, Akrout H, Thevenin N, Ruidavets L, Muller A, Bennici S (2019) The use of exhausted grape marc to produce biofuels and biofertilizers: Effect of pyrolysis temperatures on biochars properties. Renew Sustain Energy Rev 107:425–433. https://doi.org/10.1016/j.rser.2019.03.034

Ismail IS, Singh G, Smith P, Kim S, Yang J-H, Joseph S, Yusup S, Singh M, Bansal V, Talapaneni SN, Vinu A (2020) Oxygen functionalized porous activated biocarbons with high surface area derived from grape marc for enhanced capture of CO2 at elevated-pressure. Carbon 160:113–124. https://doi.org/10.1016/j.carbon.2020.01.008

Jimenez-Cordero D, Heras F, Alonso-Morales N, Gilarranz MA, Rodriguez JJ (2013) Porous structure and morphology of granular chars from flash and conventional pyrolysis of grape seeds. Biomass Bioenergy 54:123–132. https://doi.org/10.1016/j.biombioe.2013.03.020

Jin Q, Wang Z, Feng Y, Kim Y-T, Stewart AC, O’Keefe SF, Neilson AP, He Z, Huang H (2020) Grape pomace and its secondary waste management: Biochar production for a broad range of lead (Pb) removal from water. Environ Res 186:109442. https://doi.org/10.1016/j.envres.2020.109442

Khaledi S, Delbari M, Galavi H, Bagheri H, Chari MM (2023) Effects of biochar particle size, biochar application rate, and moisture content on thermal properties of an unsaturated sandy loam soil. Soil Tillage Res 226:105579. https://doi.org/10.1016/j.still.2022.105579

Libutti A, Francavilla M, Monteleone M (2021) Hydrological Properties of a Clay Loam Soil as Affected by Biochar Application in a Pot Experiment. Agronomy 11(3):489. https://doi.org/10.3390/agronomy11030489

Lin J-C, Mariuzza D, Volpe M, Fiori L, Ceylan S, Goldfarb JL (2021) Integrated thermochemical conversion process for valorizing mixed agricultural and dairy waste to nutrient-enriched biochars and biofuels. Bioresour Technol 328:124765. https://doi.org/10.1016/j.biortech.2021.124765

Manolikaki II, Mangolis A, Diamadopoulos E (2016) The impact of biochars prepared from agricultural residues on phosphorus release and availability in two fertile soils. J Environ Manag 181:536–543. https://doi.org/10.1016/j.jenvman.2016.07.012

Marshall J, Muhlack R, Morton BJ, Dunnigan L, Chittleborough D, Kwong CW (2019) Pyrolysis Temperature Effects on Biochar–Water Interactions and Application for Improved Water Holding Capacity in Vineyard Soils. Soil Syst 3(2):27. https://doi.org/10.3390/soilsystems3020027

Marshall JA, Morton BJ, Muhlack R, Chittleborough D, Kwong CW (2017) Recovery of phosphate from calcium-containing aqueous solution resulting from biochar-induced calcium phosphate precipitation. J Clean Prod 165:27–35. https://doi.org/10.1016/j.jclepro.2017.07.042

Mena IF, Diaz E, Rodriguez JJ, Mohedano AF (2017) CWPO of bisphenol A with iron catalysts supported on microporous carbons from grape seeds activation. Chem Eng J 318:153–160. https://doi.org/10.1016/j.cej.2016.06.029

Pardo R, Taboada-Ruiz L, Fuente E, Ruiz B, Díaz-Somoano M, Calvo LF, Paniagua S (2023) Exploring the potential of conventional and flash pyrolysis methods for the valorisation of grape seed and chestnut shell biomass from agri-food industry waste. Biomass Bioenergy 177:106942. https://doi.org/10.1016/j.biombioe.2023.106942

Petrova T, Naydenova I, Ribau J, Ferreira AF (2023) Biochar from Agro-Forest Residue: Application Perspective Based on Decision Support Analysis. Appl Sci 13(5):3240. https://doi.org/10.3390/app13053240

Rivelli AR, Libutti A (2022) Effect of Biochar and Inorganic or Organic Fertilizer Co-Application on Soil Properties, Plant Growth and Nutrient Content in Swiss Chard. Agronomy 12(9):2089. https://doi.org/10.3390/agronomy12092089

Sfakiotakis S, Vamvuka D (2018) Thermal decomposition behavior, characterization and evaluation of pyrolysis products of agricultural wastes. J Energy Inst 91(6):951–961. https://doi.org/10.1016/j.joei.2017.09.001

Tag AT, Duman G, Ucar S, Yanik J (2016) Effects of feedstock type and pyrolysis temperature on potential applications of biochar. J Anal Appl Pyrolysis 120:200–206. https://doi.org/10.1016/j.jaap.2016.05.006

Uysal Y, Doğaroğlu ZG, Makas MN, Çaylali Z (2024) Boosting Water Retention in Agriculture: Vine Biochar‐Doped Hydrogels’ Swelling and Germination Effects. Glob Challenges 8(5):2300254. https://doi.org/10.1002/gch2.202300254

Vamvuka D, Esser K, Komnitsas K (2020) Investigating the Suitability of Grape Husks Biochar, Municipal Solid Wastes Compost and Mixtures of Them for Agricultural Applications to Mediterranean Soils. Resources 9(3):33. https://doi.org/10.3390/resources9030033

Volpe M, Fiori L, Panno D, Volpe R, Messineo A (2018) Assessment of bio-combustibles production via slow pyrolysis of wine industry residues. AIP Conf. Proc., Thessaloniki, Greece, p 140010

Yoon J-Y, Kim JE, Song HJ, Oh KB, Jo JW, Yang Y-H, Lee SH, Kang G, Kim HJ, Choi Y-K (2021) Assessment of adsorptive behaviors and properties of grape pomace-derived biochar as adsorbent for removal of cymoxanil pesticide. Environ Technol Innov 21:101242. https://doi.org/10.1016/j.eti.2020.101242

Zabaniotou A, Kamaterou P, Pavlou A, Panayiotou C (2018) Sustainable bioeconomy transitions: Targeting value capture by integrating pyrolysis in a winery waste biorefinery. J Clean Prod 172:3387–3397. https://doi.org/10.1016/j.jclepro.2017.11.077
